# Supplementary material for: Is something rotten in the state of Denmark? Cross-national evidence for widespread involvement but not systematic use of questionable research practices across all fields of research
Source: PLoS One. 2024 Aug 12;19(8):e0304342. doi: 10.1371/journal.pone.0304342 (PMC11318862; doi:10.1371/journal.pone.0304342)
Supplement: S1 Table — (PDF) [file pone.0304342.s001.pdf]

S1 Table. QRR statements, category and wording in the questionnaire.

| No. | Categories | QRP statements wording |
|---|---|---|
| 1 | Authorship | Including authors on a publication who has not contributed sufficiently to the work to merit authorship. |
| 2 | Authorship | Failing to offer authorship to collaborators who had contributed sufficiently to the work to merit authorship. |
| 3 | Transparency | In a publication, failing to disclose relevant personal, financial, political or intellectual conflicts of interests. |
| 4 | Selective analysis | In significance testing studies, continue to collect more data in order to render non-significant results significant. For example: If initial results are not statistically significant, collect more data until you get the desired result. |
| 5 | Selective analysis | In significance testing studies, continue to reanalyse data until a statistically significant result is obtained. For example: If initial results are not statistically significant, remove or transform data; use other statistical tests; redefine the outcome variable, switching to an alternate control group; trying various combinations of independent and control variables; analysing various subgroups and so forth; thus, reanalysing data until you get the desired result. |
| 6 | Recycling | Publishing work despite knowing that it is redundant and does not contribute to the existing knowledge base. Please note, reviews, translations and other types of research publications are not necessarily redundant if they fill a scholarly need in the scientific communication. |
| 7 | Citing practices | Citing literature deemed relevant for your study without actually having read it. |
| 8 | Misleading reporting | Claim to have used a particular qualitative analytical approach appropriately, for example "grounded theory" or "triangulation", when this was not the case. |
| 9 | Transparency | Avoiding to share data, research protocols, information on experimental setup, instrumentation, coding, or other information about a study requested by colleagues to evade transparency. |
| 10 | Reviewing | Agree to review a manuscript despite knowing that you have inadequate expertise to provide a competent review. |
| 11 | Reviewing | When reviewing a manuscript, not investing the effort necessary to conduct a thorough review. |
| 12 | Reviewing | Submitting a biased review report that evaluated the manuscript unfairly. |
| 13 | Recycling; transparency | In a publication, deliberately reuse all or parts of previously published data without disclosure. |
| 14 | Recycling | Deliberately divide the results of a study over more publications than needed with the intention to increase the number of publications. |
| 15 | Citing practices | Selectively citing irrelevant or unnecessary publications to please reviewers or editors. |
| 16 | Citing practices | Deliberately cite own publications more than warranted by their relevance, to promote the visibility of your work or improve your citation metrics. |
| 17 | Citing practices | Deliberately disregard citing relevant publications that contradict own beliefs or research. |
| 18 | Selective reporting | Selectively focusing on parts of the data or source material that support your preconceptions or hypotheses and deliberately disregarding parts that do not. Source material can be of all kinds, such as interviewees, documents, primary sources, etc. |
| 19 | Selective reporting | Deliberately refrain from reporting findings that could weaken or contradict own theories, hypotheses or findings. |
| 20 | Spin | Wilfully present findings as more "clear-cut" than justified by the data. For example, neglecting to disclose contradictory results, or thoroughly discuss study limitations, or deliberately overlooking counter arguments. |
| 21 | Selective analysis | Without disclosure, formulate or change hypotheses after having seen the results, thereby presenting an unexpected finding as having been predicted from the start in the form of a research hypothesis (quantitative study). |
| 22 | Selective analysis | Without disclosure, presenting an unexpected finding as having been predicted from the start (qualitative study). |
| 23 | Misleading interpretation | Present statistically significant main findings without distinguishing between their "statistical significance" and their potential practical or theoretical importance. For example, claiming a "significant" finding solely based on the p-value and not the effect size. Notice, importance refers to clinical, economic, biological, psychological, sociological etc. |
| 24 | Misleading interpretation | Report main findings that turned out not to be "statistically significant", as evidence for no difference, no effect or no association between study groups or variables. |
| 25 | Plagiarism | Deliberately using another researcher's unpublished idea without giving credit. For example, publishing an idea voiced by a colleague at an informal meeting without giving her/him credit. |
