# Supplementary material for: Is something rotten in the state of Denmark? Cross-national evidence for widespread involvement but not systematic use of questionable research practices across all fields of research
Source: PLoS One. 2024 Aug 12;19(8):e0304342. doi: 10.1371/journal.pone.0304342 (PMC11318862; doi:10.1371/journal.pone.0304342)
Supplement: S1 File — (PDF) [file pone.0304342.s002.pdf]

S2 Table. Assignment of 25 QRP statements according to preferred research approach.

QRP No.	Category	Category ID	QRP ID	Non-empirical	Empirical quantitative	Empirical quantitative: significance	Empirical qualitative	Total eligible
1	Authorship	1	#1 (au)	1	1	1	1	4
2	Authorship	1	#2 (au)	1	1	1	1	4
3	Transparency	2	#3	1	1	1	1	4
4	Selective analysis	3	#4a (sst)	0	0	1	0	1
5	Selective analysis	3	#4b (sst)	0	0	1	0	1
6	Recycling	4	#5	1	1	1	1	4
7	Citing practices	5	#6	1	1	1	1	4
8	Misleading reporting	11	#7	0	0	0	1	1
9	Transparency	6	#8	0	1	1	1	3
10	Reviewing	9	#9(rev)	1	1	1	1	4
11	Reviewing	9	#10 (rev)	1	1	1	1	4
12	Reviewing	9	#11 (rev)	1	1	1	1	4
13	Recycling; transparency	8	#12	0	1	1	1	3
14	Recycling	8	#13	0	1	1	1	3
15	Citing practices	10	#14	1	1	1	1	4
16	Citing practices	10	#15	1	1	1	1	4
17	Citing practices	10	#16	1	1	1	1	4
18	Selective reporting	11	#17	0	1	1	1	3
19	Selective reporting	11	#18	0	1	1	1	3
20	Spin	11	#19	0	1	1	1	3
21	Selectivity analysis	11	#20	0	1	1	0	2
22	Selectivity analysis	11	#21	0	0	0	1	1
23	Misleading interpretation	12	#22(sst)	0	0	1	0	1
24	Misleading interpretation	12	#23(sst)	0	0	1	0	1
25	Plagiarism	7	#24	1	0	0	1	2
			**Total**	12	18	22	20	

1) Assignment = 1, otherwise 0

S3 Table. Assignment rules of 25 QRP statements.

	Category ID	Category	No. items	Category rule	Item rule	Items
Non-empirical	1	Authorship	2	Mandatory	Mandatory 2Q	#1; #2
	2	Transparency	1	Mandatory	Mandatory 1Q	#3
	3	Selective analysis	0	Out	n/a	n/a
	4	Recycling	1	Mandatory	Mandatory 1Q	#6
	5	Citing practices	1	Mandatory	Mandatory 1Q	#7
	2	Transparency	0	Out	n/a	n/a
	11	Plagiarism	1	Mandatory	Mandatory 1Q	#25
	4	Recycling	0	Out	n/a	n/a
	7	Reviewing	3	Mandatory	Random 1Q	#10; #11; #12
	5	Citing practices	3	Mandatory	Mandatory 3Q	#15; #16; #17
	8; 3; 9	Selective reporting; selective analysis; spin	0	Out	n/a	n/a
	10	Misleading interpretation	0	Out	n/a	n/a
Empirical quantitative	1	Authorship	2	Random	Max 1Q	#1; #2
	2	Transparency	1	Random	Random	#3
	3	Selective analysis	0	Out	n/a	n/a
	4	Recycling	1	Random	Random	#6
	5	Citing practices	1	Random	Random	#7
	2	Transparency	1	Random	Random	#9
	11	Plagiarism	0	Out	n/a	n/a
	4	Recycling	2	Random	Random	#13; #14
	7	Reviewing	3	Random	Max 2Q	#10; #11; #12
	5	Citing practices	3	Random	Random	#15; #16; #17
	8; 3; 9	Selective reporting; selective analysis; spin	4	Mandatory	Mandatory: 1Q + Min Random: 2Q	Mandatory: #21 + #18; #19; #20
	10	Misleading interpretation	0	Out	n/a	
Empirical quantitative: significance	1	Authorship	2	Random	Max 1Q	#1; #2
	2	Transparency	1	Random	Random	#3
	3	Selective analysis	2	Mandatory	Mandatory 2Q	#4; #5 Always in pair
	4	Recycling	1	Random	Random	#6
	5	Citing practices	1	Random	Random	#7
	2	Transparency	1	Random	Random	#9
	11	Plagiarism	0	Out	n/a	
	4	Recycling	2	Random	Random	#13; #14
	7	Reviewing	3	Random	Max 1Q	#10; #11; #12
	5	Citing practices	3	Random	Random	#15; #16; #17
	8; 3; 9	Selective reporting; selective analysis; spin	4	Mandatory IF both cat 2 Qs > 0	Mandatory 1Q IF both cat 2 Qs > 0 + Min 1Q	
	8; 3; 9	Selective reporting; selective analysis; spin	4	Random IF both cat 2 Qs = 0	Min 2Q	#18; #19; #20 - #21 is OUT
	10	Misleading interpretation	2	Mandatory IF both cat 2 Qs > 0	Random 1Q	#23; #24
	10	Misleading interpretation	2	OUT IF both cat 2 Qs = 0	n/a	n/a

Empirical qualitative	1	Authorship	2	Random	Max 1Q	#1(au); #2(au)
	2	Transparency	1	Random	Random	#3
	3	Selective analysis	0	Out	n/a	n/a
	4	Recycling	1	Random	Random	#6
	5	Citing practices	1	Random	Random	#7
	2	Transparency	1	Random	Random	#9
	11	Plagiarism	1	Random	Random	#25
	4	Recycling	2	Random	Random	#13; #14
	7	Reviewing	3	Random	Max 2Q	#10(rev); #11(rev); #12(rev)
	5	Citing practices	3	Random	n/a	n/a
	8; 3; 9	Selective reporting; selective analysis; spin	5	Mandatory	Mandatory 2Q + Random 2Q	Mandatory #8; #22 + #15; #1; #18; #19
	10	Misleading interpretation	0	Out	n/a	n/a

1) Q = QRP statement (item)
