# Supplementary material for: Is something rotten in the state of Denmark? Cross-national evidence for widespread involvement but not systematic use of questionable research practices across all fields of research
Source: PLoS One. 2024 Aug 12;19(8):e0304342. doi: 10.1371/journal.pone.0304342 (PMC11318862; doi:10.1371/journal.pone.0304342)
Supplement: S2 File — (PDF) [file pone.0304342.s003.pdf]

S4 Table: Statements concerning the research conditions.

Perceived ‘pressure’ (6 items) (rs1)

In the following three pages we pose some statements related to the context and conditions under which we pursue our research<br>**Please indicate the extent to which you agree with the following statements, as they relate to your particular context and conditions**.
7-point Likert scales: Strongly disagree -- Neutral -- Strongly agree
#1: My field of research is influenced by external interests (e.g., financial interests, high political stakes and/or high public attention).
#2: I feel a strong pressure to attract external funding.
#3: When applying for funding I feel a pressure to oversell the potential impact of my projects.
#4: If two equally talented researchers apply for funding, it will be awarded to the person with highest research integrity.
#5: I feel a pressure to publish in a prestigious outlet.
#6: Bibliometric indicators are important for my career (e.g., citations, H-index, Journal Impact Factors etc.)

‘Peer review’ conditions (4 items) (rs2)

**Please indicate the extent to which you agree with the following statements, as they relate to your particular context and conditions**.
7-point Likert scales: Strongly disagree -- Neutral -- Strongly agree
#1: The peer review and editorial processes determining publication decisions encourage me to oversell my results.
#2: The peer review and editorial processes determining publication decisions provide a sufficient safeguard against questionable research practices within my field.
#3: Questionable research practices are rewarded in the leading publication outlets within my field.
#4: The most prestigious publication outlets in my field are those that publish research with the highest standard of integrity

Perceived ‘local research culture’ (6 items) (rs3)

**Please indicate the extent to which you agree with the following statements, as they relate to your particular context and conditions**.
7-point Likert scales: Strongly disagree -- Neutral -- Strongly agree
#1: The reward system at my department or center encourages me to carry out the most rigorous and solid research possible.
#2: The local leadership at my department or center are role models in terms of upholding research integrity
#3: The most prominent researchers at my department of center are role models in terms of upholding research integrity
#4: If I conduct my research without compromising research integrity, it will have negative effects on my career.
#5: If two equally talented researchers apply for a position, it will be offered to the person with the highest integrity.
#6: The peer culture within my department or center functions as a safeguard against questionable research practices.

S5 Figure: Distribution of responses to the items concerning the research conditions.

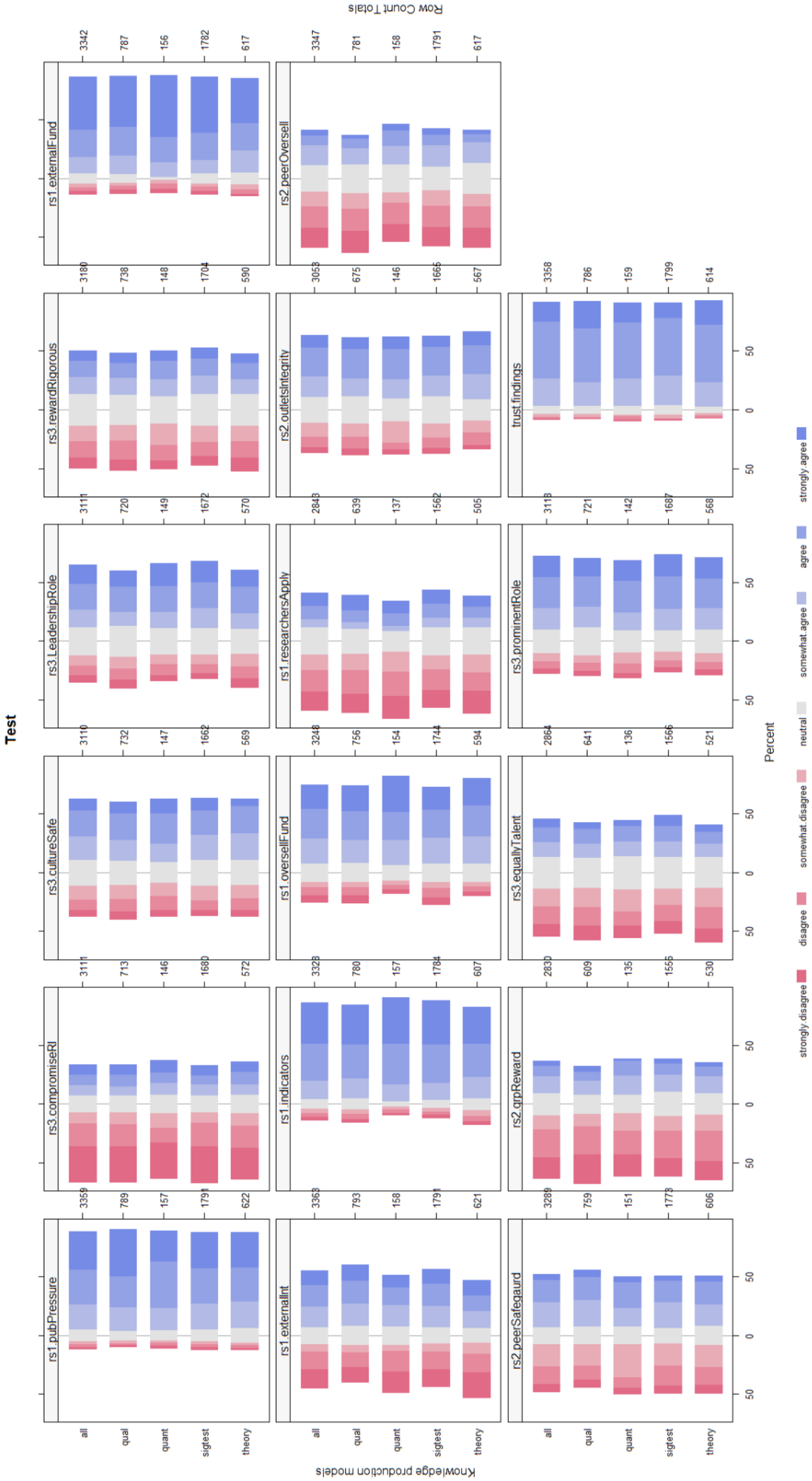

S6 Figure: Correlation matrix of predictors in the Danish survey.

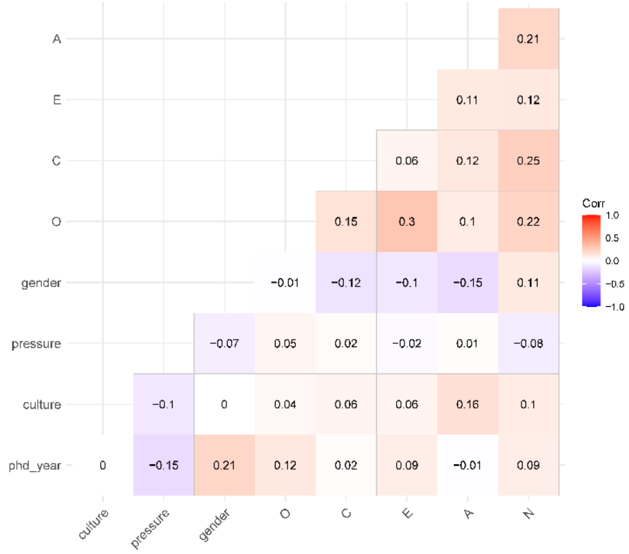

S7 Figure: Correlation matrix of predictors in the international survey.

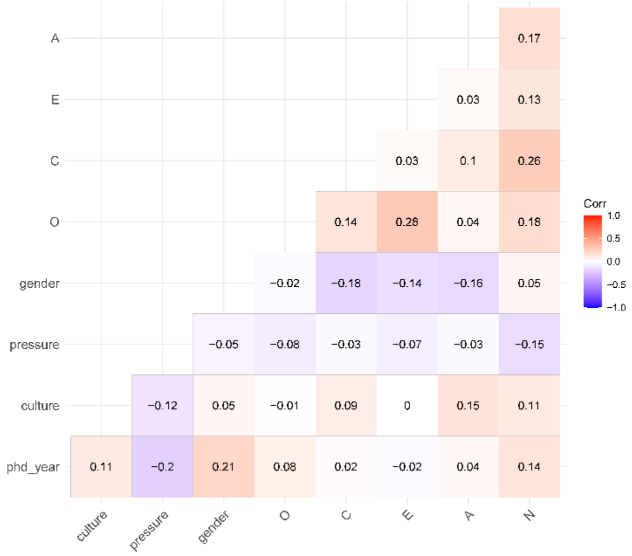

S8 Figure: Correlation matrix of predictors and QRPs in the Danish survey.

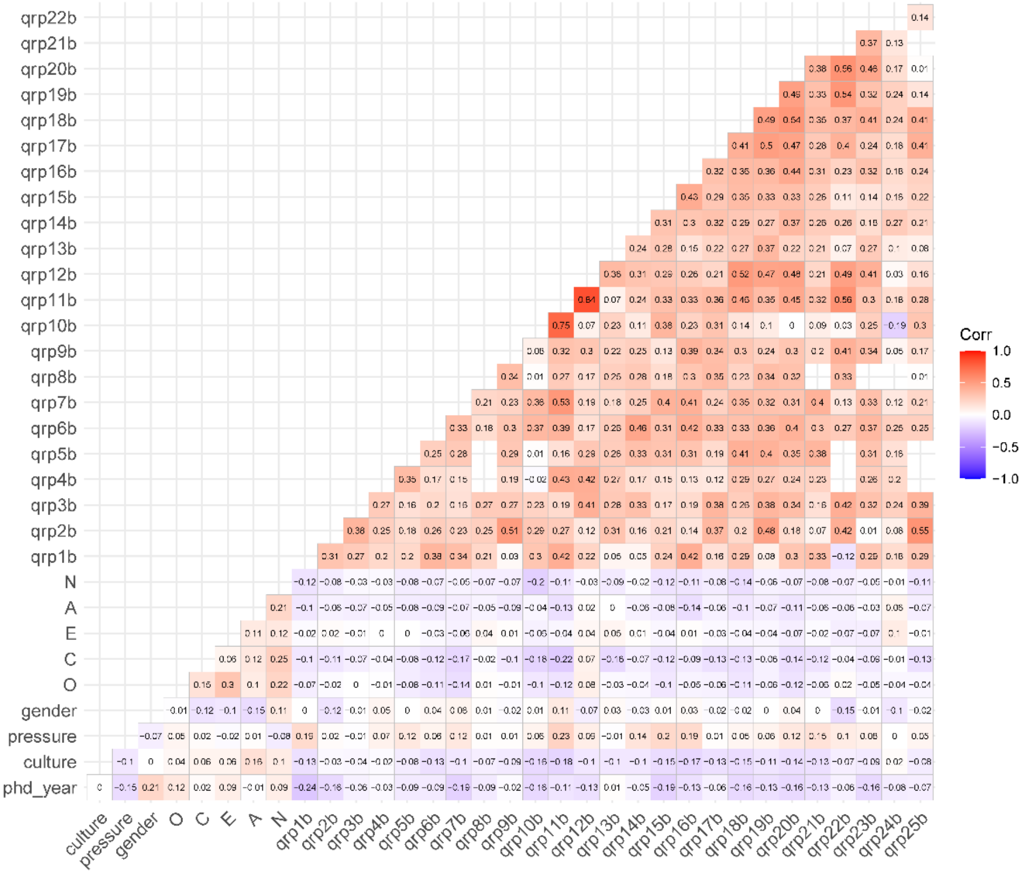

S9 Figure: Correlation matrix of predictors and QRPs in the international survey.

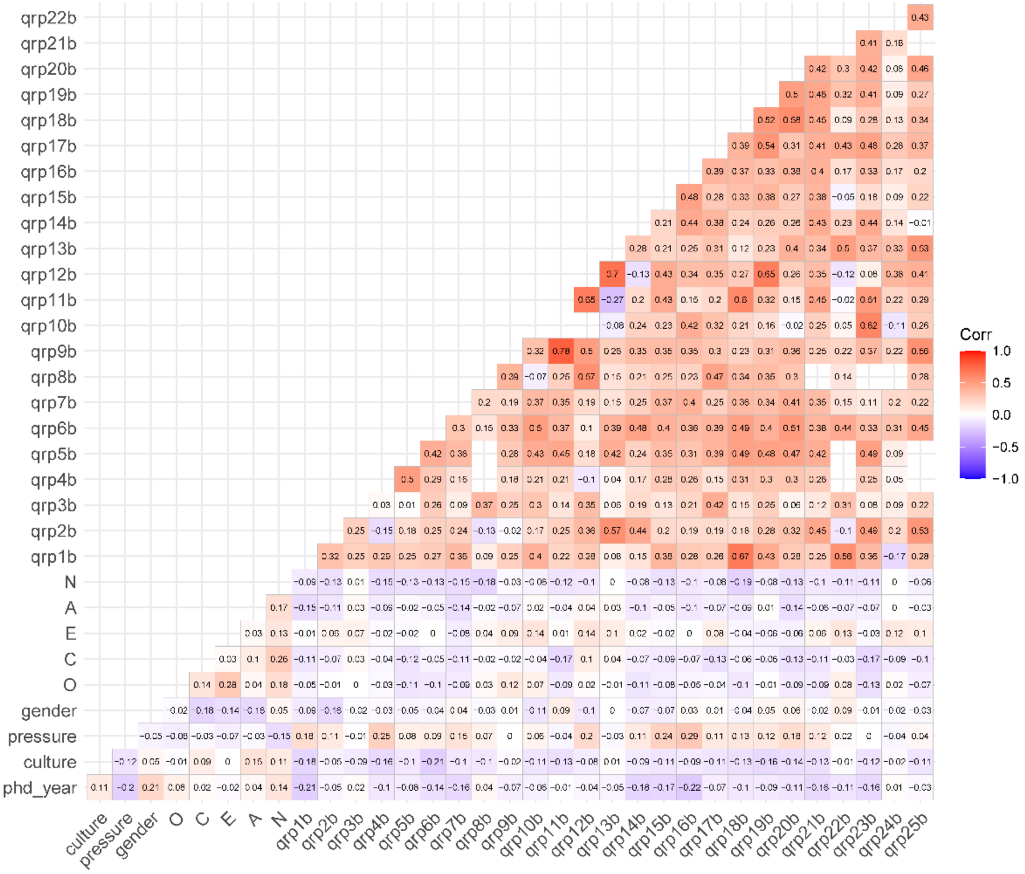

In the manuscript we report regression using a Bayesian implementation of the Linear Probability Model (LPM) [1]. As LPM uses ordinary least squares on a bounded outcome variable, we compared findings from the LPM models with fractional regressions [2]. As results are similar, we report probability changes in the predictors from the LPM models in the manuscript, as they are more intuitive to interpret than log odds from fractional regressions. Here we present the results for the fractional regressions.

We examine potential associations of social, individual and demographic factors with self-reported prevalence with QRPs using a Bayesian implementation of fractional regression. The outcome y is the overall self-reported prevalence with QRPs for the individual respondent measured as a proportion in the interval [0, 1]. As the proportions are based on individual scores in the nominator and total values in the denominator, individually adjusted for the number of QRP statements the respondent answered (max = 9) , we are able to use standard logistic regression with the binomial distribution (Papke & Wooldridge, 1996):

$$\mathcal{L} \sim y(\ln g(X\beta)) + (1-y)(1-\ln g(X\beta),$$

where y is our outcome variable, Xβ are the linear predictors, and g(·) is the link function in our case the logit. Notice, the nonlinear link function ensures that predictions lie inside the unit interval. The predictors include five individual factors (the Big Five: 'OCEAN'), two social factors ('pressure' and 'local research culture'), as well as two demographic factors (gender and academic age, the latter is the time from PhD to survey year). The Bayesian models were implemented in R (Team, 2021) using the brms package (v 2.16.1) (Bürkner, 2017) using weakly informative priors, N(0,10). Log odds outcomes from the models are exponentiated and reported as odds ratios (OR) with posterior 95% credible intervals.

S10 Table. Results of fractional regressions. Danish response patterns: Non-empirical (compare to Fig 5 in manuscript).

	tot resp|trials (max resp)		tot resp|trials (max resp)	
Predictors	Log-Odds	CI (95%)	Odds Ratios	CI (95%)
Intercept	0.03	-0.34 – 0.39	1.03	0.71 – 1.48
Openness	-0.09	-0.13 – -0.06	0.91	0.88 – 0.94
Conscientiousness	-0.18	-0.22–-0.15	0.83	0.81–0.86
Extraversion	0.02	-0.00–0.04	1.02	1.00–1.05
Agreeableness	-0.1	-0.13–-0.06	0.91	0.88–0.94
Neuroticism	-0.07	-0.10–-0.04	0.93	0.90–0.96
Gender (ref=not male)	0.33	0.24–0.43	1.4	1.27–1.54
Year after PhD	-0.02	-0.03–-0.02	0.98	0.97–0.98
Local culture	-0.16	-0.18–-0.13	0.85	0.83–0.88
Perceived pressure	0.18	0.15–0.22	1.2	1.16–1.24
Observations	554			
R2Bayes	0.226			

S11 Table. Results of fractional regressions. Danish response patterns: Qualitative (compare to Fig5 in manuscript).

	tot resp|trials (max resp)		tot resp|trials (max resp)	
Predictors	Log-Odds	CI (95%)	Odds Ratios	CI (95%)
Intercept	-1.04	-1.36–-0.70	0.35	0.26–0.50
Openness	-0.01	-0.05–0.02	0.99	0.95–1.02
Conscientiousness	-0.11	-0.14–-0.09	0.89	0.87–0.92
Extraversion	0.01	-0.01–0.04	1.01	0.99–1.04
Agreeableness	-0.08	-0.12–-0.05	0.92	0.89–0.95
Neuroticism	-0.03	-0.05–-0.00	0.97	0.95–1.00
Gender (ref=not male)	0.1	0.03–0.16	1.1	1.03–1.18
Year after PhD	-0.02	-0.02–-0.01	0.98	0.98–0.99
Local culture	-0.13	-0.16–-0.11	0.88	0.85–0.90
Perceived pressure	0.16	0.13–0.20	1.18	1.14–1.22
Observations	681			
R2Bayes	0.118			

S12 Table. Results of fractional regressions. Danish response patterns: Quantitative (compare to Fig5 in manuscript).

	tot resp|trials (max resp)		tot resp|trials (max resp)	
Predictors	Log-Odds	CI (95%)	Log-Odds	CI (95%)
Intercept	-1.56	-2.33–-0.83	0.21	0.10–0.44
Openness	-0.01	-0.08–0.07	0.99	0.93–1.07
Conscientiousness	-0.18	-0.24–-0.13	0.83	0.79–0.88
Extraversion	0.01	-0.03–0.06	1.01	0.97–1.06
Agreeableness	0.09	0.02–0.16	1.09	1.02–1.17
Neuroticism	-0.03	-0.10–0.03	0.97	0.91–1.03
Gender (ref=not male)	-0.02	-0.20–0.16	0.98	0.82–1.17
Year after PhD	-0.03	-0.03–-0.02	0.98	0.97–0.98
Local culture	-0.23	-0.29–-0.18	0.79	0.75–0.84
Perceived pressure	0.31	0.23–0.39	1.36	1.26–1.48
Observations	141			
R2Bayes	0.227			

S13 Table. Results of fractional regressions. Danish response patterns: Quantitative (sig test) (compare to Fig 5in manuscript).

	tot resp|trials (max resp)		tot resp|trials (max resp)	
Predictors	Log-Odds	CI (95%)	Log-Odds	CI (95%)
Intercept	-0.66	-0.87–-0.46	0.52	0.42–0.63
Openness	-0.08	-0.10–-0.06	0.92	0.90–0.94
Conscientiousness	-0.1	-0.12–-0.08	0.91	0.89–0.92
Extraversion	0.02	0.01–0.04	1.03	1.01–1.04

Agreeableness	-0.06	-0.08–-0.04	0.94	0.92–0.96
Neuroticism	-0.01	-0.03–0.00	0.99	0.97–1.00
Gender (ref=not male)	0.08	0.03–0.12	1.08	1.03–1.13
Year after PhD	-0.02	-0.02–-0.02	0.98	0.98–0.98
Local culture	-0.11	-0.13–-0.09	0.9	0.88–0.91
Perceived pressure	0.13	0.12–0.15	1.14	1.12–1.17
Observations	1624			
R2Bayes	0.105			

S14 Table. Results of fractional regressions. International response patterns: Non-empirical (compare to Fig6 in manuscript).

	tot resp|trials (max resp)		tot resp|trials (max resp)	
Predictors	Log-Odds	CI (95%)	Log-Odds	CI (95%)
Intercept	-1.74	-2.26–-1.26	0.18	0.10–0.28
Openness	-0.09	-0.14–-0.04	0.91	0.87–0.96
Conscientiousness	-0.09	-0.13–-0.05	0.91	0.88–0.95
Extraversion	0.08	0.05–0.11	1.08	1.05–1.12
Agreeableness	-0.06	-0.10–-0.02	0.94	0.90–0.98
Neuroticism	-0.06	-0.10–-0.03	0.94	0.90–0.97
Gender (ref=not male)	-0.02	-0.13–0.10	0.98	0.88–1.11
Year after PhD	-0.01	-0.02–-0.01	0.99	0.98–0.99
Local culture	-0.03	-0.07–0.00	0.97	0.93–1.00
Perceived pressure	0.29	0.24–0.33	1.33	1.27–1.40
Observations	283			
R2Bayes	0.206			

S15 Table. Results of fractional regressions. International response patterns: Qualitative (compare to Fig6 in manuscript).

	tot resp|trials (max resp)		tot resp|trials (max resp)	
Predictors	Log-Odds	CI (95%)	Log-Odds	CI (95%)
Intercept	-2.04	-2.57–-1.53	0.13	0.08–0.22
Openness	0.09	0.04–0.15	1.1	1.04–1.16
Conscientiousness	-0.01	-0.06–0.04	0.99	0.95–1.04
Extraversion	0.09	0.06–0.13	1.1	1.06–1.14
Agreeableness	-0.05	-0.10–-0.00	0.95	0.91–1.00
Neuroticism	-0.15	-0.19–-0.10	0.86	0.83–0.90
Gender (ref=not male)	0.2	0.09–0.30	1.22	1.09–1.36
Year after PhD	-0.01	-0.01–-0.00	0.99	0.99–1.00
Local culture	-0.08	-0.11–-0.04	0.93	0.89–0.96
Perceived pressure	0.11	0.06–0.16	1.11	1.06–1.17
Observations	242			

R2Bayes	0.121		

S16 Table. Results of fractional regressions. International response patterns: Quantitative (compare to Fig6 in manuscript).

	tot resp|trials (max resp)		tot resp|trials (max resp)	
Predictors	Log-Odds	CI (95%)	Log-Odds	CI (95%)
Intercept	0.52	-0.75–1.79	1.68	0.47–5.99
Openness	0.08	-0.05–0.22	1.08	0.95–1.25
Conscientiousness	-0.01	-0.12–0.09	0.99	0.89–1.10
Extraversion	-0.31	-0.42–-0.20	0.73	0.66–0.82
Agreeableness	-0.04	-0.16–0.09	0.96	0.85–1.09
Neuroticism	-0.06	-0.15–0.04	0.95	0.86–1.04
Gender (ref=not male)	-0.28	-0.61–0.08	0.76	0.54–1.08
Year after PhD	-0.03	-0.04–-0.02	0.97	0.96–0.98
Local culture	-0.25	-0.39–-0.11	0.78	0.68–0.90
Perceived pressure	0.07	-0.05–0.20	1.07	0.95–1.23
Observations	44			
R2Bayes	0.294			

S17 Table. Results of fractional regressions. International response patterns: Quantitative (sig test) (compare to Fig6 in manuscript).

	tot resp|trials (max resp)		tot resp|trials (max resp)	
Predictors	Log-Odds	CI (95%)	Log-Odds	CI (95%)
Intercept	0.08	-0.20–0.37	1.09	0.82–1.44
Openness	-0.07	-0.10–-0.04	0.93	0.91–0.96
Conscientiousness	-0.1	-0.13–-0.08	0.9	0.88–0.93
Extraversion	0.01	-0.01–0.03	1.01	0.99–1.03
Agreeableness	-0.04	-0.06–-0.01	0.96	0.94–0.99
Neuroticism	-0.05	-0.07–-0.03	0.95	0.93–0.97
Gender (ref=not male)	-0.03	-0.09–0.02	0.97	0.91–1.03
Year after PhD	-0.01	-0.01–-0.01	0.99	0.99–0.99
Local culture	-0.1	-0.12–-0.09	0.9	0.88–0.92
Perceived pressure	0.1	0.07–0.12	1.1	1.07–1.13
Observations	616			
R2Bayes	0.129			

S18 Table: Descriptive statistics. Danish response patterns: Preferred research approaches.

**Non-empirical**					n = 589
	**Mean**	**Std.Dev**	**Min**	**Median**	**Max**
Openness	5.6	1.0	1.5	6	7
Conscientiousness	5.5	1.1	2	5.5	7
Extraversion	4.4	1.5	1	4.5	7
Agreeableness	4.5	1.1	1	4.5	7
Neuroticism	5.2	1.3	1	5.5	7
Year since PhD	13.8	10.4	-1	12	53
Local culture	4.4	1.3	1	4.5	7
Perceived pressure	5.5	1.0	1	5.5	7
Gender (ref=not male)					
0	104	18%			
1	458	78%			
Missing	27	5%			
	589				

**Qualitative**					n = 722
	**Mean**	**Std.Dev**	**Min**	**Median**	**Max**
Openness	5.6	1.0	1	6	7
Conscientiousness	5.7	1.1	1.5	6	7
Extraversion	4.8	1.5	1	5	7
Agreeableness	4.6	1.1	1	4.5	7
Neuroticism	5.2	1.3	1	5.5	7
Year since PhD	12.5	9.7	-1	11	58
Local culture	4.4	1.3	1	4.5	7
Perceived pressure	5.5	1.1	1	5.75	7
Gender (ref=not male)					
0	360	50%			
1	331	46%			
Missing	31	4%			
	722				

**Quantitative**					n = 151
	**Mean**	**Std.Dev**	**Min**	**Median**	**Max**
Openness	5.5	1.0	3	5.5	7
Conscientiousness	5.6	1.1	2	6	7
Extraversion	4.4	1.6	1	4.5	7
Agreeableness	4.4	1.0	1	4.5	7
Neuroticism	5.3	1.2	1.5	5.5	7
Year since PhD	10.6	10.0	-1	6.5	46
Local culture	4.6	1.4	1	4.75	7
Perceived pressure	5.7	1.0	1.5	6	7
Gender (ref=not male)		Percent			
0	29	19%			
1	114	75%			
Missing	8	5%			
	151				

**Quantitative (sig test)**					n = 1,702
	**Mean**	**Std.Dev**	**Min**	**Median**	**Max**
Openness	5.6	1.0	1	6	7
Conscientiousness	5.7	1.0	1.5	6	7
Extraversion	4.6	1.5	1	4.5	7
Agreeableness	4.5	1.0	1.5	4.5	7
Neuroticism	5.3	1.2	1	5.5	7
Year since PhD	12.9	10.3	-1	11	58
Local culture	4.6	1.3	1	4.75	7
Perceived pressure	5.5	1.1	1	5.75	7
Gender (ref=not male)					
0	562	33%			
1	1078	63%			
Missing	62	4%			
	1,702				

S19 Table: Descriptive statistics. International response patterns: Preferred research approaches.

**Non-empirical**					n = 299
	**Mean**	**Std.Dev**	**Min**	**Median**	**Max**
Openness	5.55	1.08	2	5.5	7
Conscientiousness	5.41	1.18	1.5	5.5	7
Extraversion	3.97	1.57	1	4	7
Agreeableness	4.54	1.17	1	4.5	7
Neuroticism	4.97	1.30	1	5	7
Year since PhD	16.68	13.71	0	13	58
Local culture	4.38	1.44	1	4.5	7
Percieved pressure	4.74	1.30	1	5	7
Gender (ref=not male)					
0	81	27			
1	202	68			
Missing	16	5			
	299				

**Qualitative**					n = 253
	**Mean**	**Std.Dev**	**Min**	**Median**	**Max**
Openness	5.7	1.1	1.5	6	7
Conscientiousness	5.7	1.1	2	6	7
Extraversion	4.5	1.6	1	4.5	7
Agreeableness	4.7	1.2	1.5	4.5	7
Neuroticism	5.2	1.3	1	5.5	7
Year since PhD	13.6	11.6	0	11	58
Local culture	4.1	1.5	1	4.25	7
Percieved pressure	5.0	1.2	1	5.2	7
Gender (ref=not male)					
0	121	48			
1	121	48			
Missing	11	4			
	253				

**Quantitative**					n = 48
	**Mean**	**Std.Dev**	**Min**	**Median**	**Max**
Openness	5.2	1.0	2.5	5.25	7
Conscientiousness	5.2	1.3	2	5.5	7
Extraversion	3.7	1.5	1	3.5	7
Agreeableness	4.4	1.3	1.5	4.25	7
Neuroticism	5.0	1.5	1.5	5	7
Year since PhD	15.3	14.0	0	11	58
Local culture	4.2	1.0	2.25	4.25	6
Percieved pressure	5.4	1.1	2.6	5.6	7
Gender (ref=not male)					
0	7	15			
1	37	77			
Missing	4	8			
	48				

**Quantitative (sig test)**					n = 636
	**Mean**	**Std.Dev**	**Min**	**Median**	**Max**
Openness	5.6	1.0	2	5.5	7
Conscientiousness	5.6	1.1	1.5	6	7
Extraversion	4.5	1.6	1	4.5	7
Agreeableness	4.6	1.1	1	4.5	7
Neuroticism	5.1	1.3	1	5.5	7
Year since PhD	14.6	12.2	0	11	56
Local culture	4.3	1.5	1	4.375	7
Percieved pressure	5.1	1.2	1.6	5.4	7
Gender (ref=not male)					
0	283	44			
1	333	52			
Missing	20	3			
	636				

S20 Table. Questionable Research Practices (QRPs): Perceived prevalence for the Danish respondents

No.	QRP	Distribution	Descriptive statistics	
1	Honorary authorships	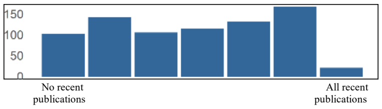	N=765<br>Admitted=666<br>Weighted mean =3.2	SD=<br>Proportion (no/yes) =0.13, 0.87<br>POMP=0.53
2	Fail to offer deserved authorship to collaborators	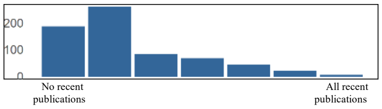	N= 644<br>Admitted=461<br>Weighted mean =1.9	SD=1.8<br>Proportion (no/yes) =0.28, 0.72<br>POMP=0.32
3	Not disclosing relevant conflicts of interests	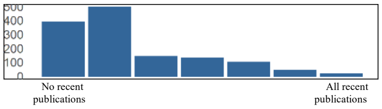	N=1,293<br>Admitted=906<br>Weighted mean =2	SD=1.9<br>Proportion (no/yes) =0.30, 0.70<br>POMP=0.33
4	Collect more data if results are non-significant	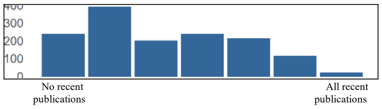	N=1,384<br>Admitted=1,150<br>Weighted mean =2.6	SD=2.5<br>Proportion (no/yes) =0.17, 0.83<br>POMP=0.43
5	Undisclosed data dredging, p-hacking	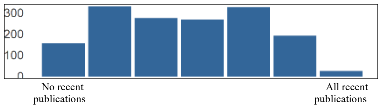	N=1,489<br>Admitted=1,345<br>Weighted mean =3	SD=2.8<br>Proportion (no/yes) =0.10, 0.90<br>POMP=0.5
6	Deliberately publishing redundant work	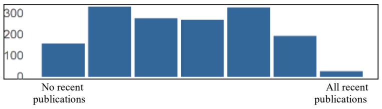	N=1,534<br>Admitted=1,385<br>Weighted mean =2.9	SD=2.7<br>Proportion (no/yes) =0.10, 0.90<br>POMP=0.48
7	Cite literature without reading read it	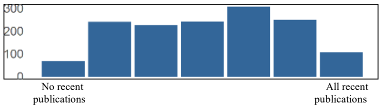	N=1,410<br>Admitted=1,347<br>Weighted mean =3.3	SD=3.2<br>Proportion (no/yes) =0.04, 0.96<br>POMP=0.55
8	Claim to have used a qualitative approach appropriately, when this was not the case	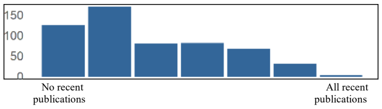	N=540<br>Admitted=417<br>Weighted mean =2.3	SD=2.2<br>Proportion (no/yes) =0.23, 0.77<br>POMP=0.38
9	Avoid to share data, code, protocol etc. requested by colleagues	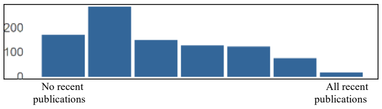	N=913<br>Admitted=748<br>Weighted mean =2.4	S<br>D=2.4<br>Proportion (no/yes) =0.18, 0.82<br>POMP=0.41
10	Agree to review a manuscript knowing that you have inadequate expertise	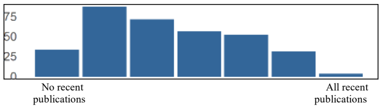	N=326<br>Admitted=294<br>Weighted mean =2.6	SD=2.4<br>Proportion (no/yes) =0.10, 0.90<br>POMP=0.43
11	Lack of sufficient effort when reviewing	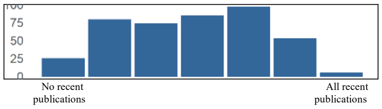	N=411<br>Admitted=387<br>Weighted mean =3	SD=2.8<br>Proportion (no/yes) =0.06, 0.94<br>POMP=0.49
12	Submitted a biased review report that evaluated the manuscript unfairly	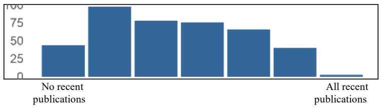	N=391<br>Admitted=349<br>Weighted mean =2.6	SD=2.5<br>Proportion (no/yes) =0.11, 0.89<br>POMP=0.44

13	Reuse previously published data without disclosure	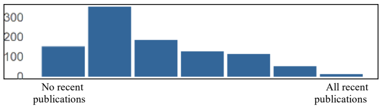	N=955<br>Admitted=809<br>Weighted mean =2.2	S<br>D=2.1<br>Proportion (no/yes) =0.15, 0.85<br>POMP=0.36
14	Salami-slicing publications	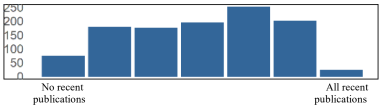	N=1,079<br>Admitted=1,007<br>Weighted mean =3.2	SD=3<br>Proportion (no/yes) =0.07, 0.93<br>POMP=0.53
15	Cite irrelevant literature to please	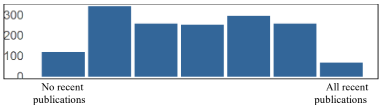	N=1,549<br>Admitted=1,437<br>Weighted mean =3	SD=2.9<br>Proportion (no/yes) =0.07, 0.93<br>POMP=0.51
16	Selective over-citing of own publications	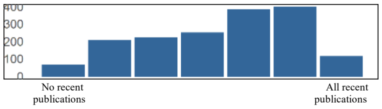	N=1,609<br>Admitted=1,548<br>Weighted mean =3.6	SD=3.4<br>Proportion (no/yes) =0.04, 0.96<br>POMP=0.59
17	Disregard citing relevant contradictory works	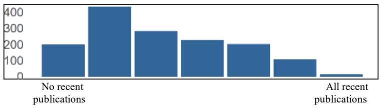	N=1,421<br>Admitted=1,227<br>Weighted mean =2.4	SD=2.3<br>Proportion (no/yes) =0.14, 0.86<br>POMP=0.4
18	Cherry-pick what supports a hypotheses and disregard that which does not	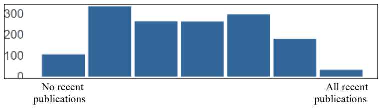	N=1,431<br>Admitted=1,332<br>Weighted mean =2.8	SD=2.7<br>Proportion (no/yes) =0.07, 0.93<br>POMP=0.47
19	Refrain from reporting findings that could weaken or contradict your findings	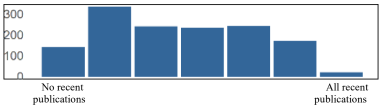	N=1,347<br>Admitted=1,212<br>Weighted mean =2.8	SD=2.6<br>Proportion (no/yes) =0.10, 0.90<br>POMP=0.46
20	Overselling results	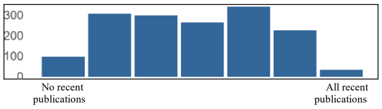	N=1,520<br>Admitted=1,429<br>Weighted mean =3	SD=2.8<br>Proportion (no/yes) =0.06, 0.94<br>POMP=0.49
21	HARKing in confirmatory quantitative studies	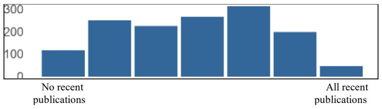	N=1,379<br>Admitted=1,267<br>Weighted mean =3.1	SD=2.9<br>Proportion (no/yes) =0.08, 0.92<br>POMP=0.51
22	HARKing in confirmatory qualitative studies	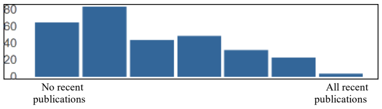	N=287<br>Admitted=224<br>Weighted mean =2.4	SD=2.3<br>Proportion (no/yes) =0.22, 0.78<br>POMP=0.4
23	Do not distinguish between statistical and practical significance	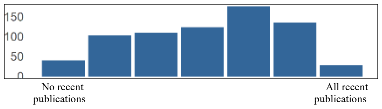	N=695<br>Admitted=658<br>Weighted mean =3.3	SD=3.1<br>Proportion (no/yes) =0.05, 0.95<br>POMP=0.55
24	Report non-significant findings as evidence for no effect	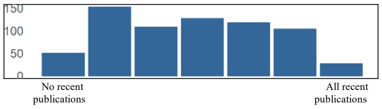	N=676<br>Admitted=627<br>Weighted mean =3	SD=2.9<br>Proportion (no/yes) =0.07, 0.93<br>POMP=0.5
25	Plagiarizing other researchers' unpublished ideas	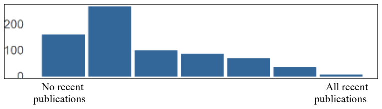	N=690<br>Admitted=534<br>Weighted mean =2.1	SD=2<br>Proportion (no/yes) =0.23, 0.77<br>POMP=0.35

*N = number of statements; Admitted = non-zero answers; weighted mean = mean of non-zero answers (1-6); sd = standard deviation of weighted mean; proportion = zero and non-zero answers; POMP = Converting scores to the Percent Of Maximum Possible score [(observed score - minimum score on the scale)/(maximum score on the scale - minimum score on the scale)].

S21 Table. Questionable Research Practices (QRPs): Perceived prevalence for the international respondents

No.	QRP	Distribution	Descriptive statistics	
1	Honorary authorships	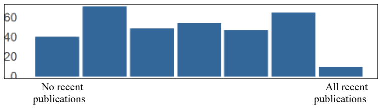	N=328<br>Admitted=289<br>Weighted mean =2.7	SD=3<br>Proportion (no/yes) =0.12, 0.88<br>POMP=0.45
2	Fail to offer deserved authorship to collaborators	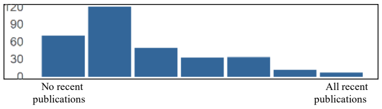	N=307<br>Admitted=239<br>Weighted mean =2.1	SD=2<br>Proportion (no/yes) =0.22, 0.78<br>POMP=0.35
3	Not disclosing relevant conflicts of interests	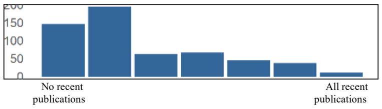	N=541<br>Admitted=397<br>Weighted mean =2.2	SD=2.2<br>Proportion (no/yes) =0.27, 0.73<br>POMP=0.37
4	Collect more data if results are non-significant	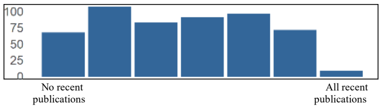	N=516<br>Admitted=450<br>Weighted mean =2.9	SD=2.8<br>Proportion (no/yes) =0.13, 0.87<br>POMP=0.49
5	Undisclosed data dredging, p-hacking	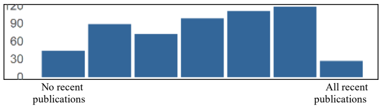	N=545<br>Admitted=503<br>Weighted mean =3.3	SD=3.2<br>Proportion (no/yes) =0.08, 0.92<br>POMP=0.56
6	Deliberately publishing redundant work	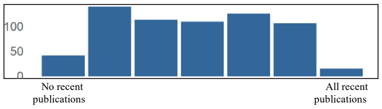	N=637<br>Admitted=598<br>Weighted mean =3	SD=2.8<br>Proportion (no/yes) =0.06, 0.94<br>POMP=0.49
7	Cite literature without reading read it	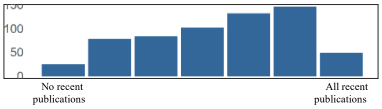	N=599<br>Admitted=577<br>Weighted mean =3.6	SD=3.4<br>Proportion (no/yes) =0.04, 0.96<br>POMP=0.59
8	Claim to have used a qualitative approach appropriately, when this was not the case	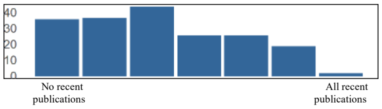	N=183<br>Admitted=148<br>Weighted mean =2.7	SD=2.5<br>Proportion (no/yes) =0.19, 0.81<br>POMP=0.44
9	Avoid to share data, code, protocol etc. requested by colleagues	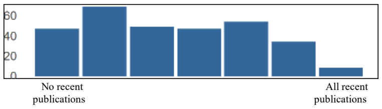	N=301<br>Admitted=255<br>Weighted mean =2.8	SD=2.7<br>Proportion (no/yes) =0.15, 0.85<br>POMP=0.47
10	Agree to review a manuscript knowing that you have inadequate expertise	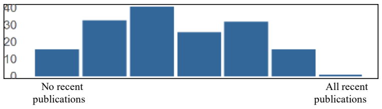	N=158<br>Admitted=143<br>Weighted mean =2.7	SD=2.5<br>Proportion (no/yes) =0.09, 0.91<br>POMP=0.45
11	Lack of sufficient effort when reviewing	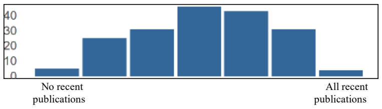	N=178<br>Admitted=174<br>Weighted mean =3.2	SD=3<br>Proportion (no/yes) =0.02, 0.98<br>POMP=0.53
12	Submitted a biased review report that evaluated the manuscript unfairly	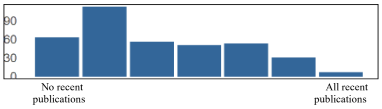	N=155<br>Admitted=143<br>Weighted mean =2.8	SD=2.6<br>Proportion (no/yes) =0.08, 0.92<br>POMP=0.47

13	Reuse previously published data without disclosure	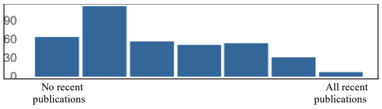	N=360<br>Admitted=299<br>Weighted mean =2.5	SD=2.4<br>Proportion (no/yes) =0.17, 0.83<br>POMP=0.42
14	Salami-slicing publications	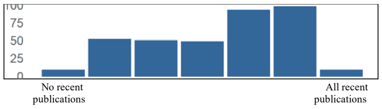	N=353<br>Admitted=345<br>Weighted mean =3.5	SD=3.3<br>Proportion (no/yes) =0.02, 0.98<br>POMP=0.58
15	Cite irrelevant literature to please	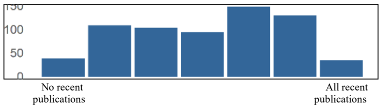	N=639<br>Admitted=604<br>Weighted mean =3.3	SD=3.2<br>Proportion (no/yes) =0.05, 0.95<br>POMP=0.55
16	Selective over-citing of own publications	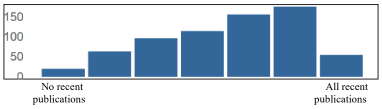	N=656<br>Admitted=639<br>Weighted mean =3.7	SD=3.5<br>Proportion (no/yes) =0.03, 0.97<br>POMP=0.61
17	Disregard citing relevant contradictory works	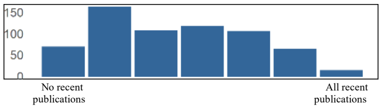	N=628<br>Admitted=561<br>Weighted mean =2.7	SD=2.6<br>Proportion (no/yes) =0.11, 0.89<br>POMP=0.45
18	Cherry-pick what supports a hypotheses and disregard that which does not	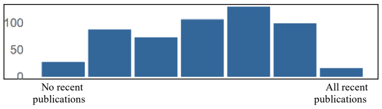	N=522<br>Admitted=497<br>Weighted mean =3.2	SD=3.1<br>Proportion (no/yes) =0.05, 0.95<br>POMP=0.54
19	Refrain from reporting findings that could weaken or contradict your findings	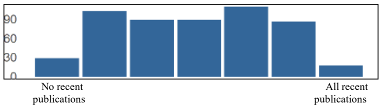	N=510<br>Admitted=483<br>Weighted mean =3.1	SD=2.9<br>Proportion (no/yes) =0.05, 0.95<br>POMP=0.51
20	Overselling results	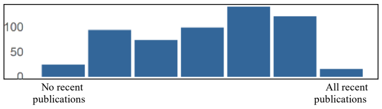	N=553<br>Admitted=532<br>Weighted mean =3.3	SD=3.1<br>Proportion (no/yes) =0.04, 0.96<br>POMP=0.55
21	HARKing in confirmatory quantitative studies	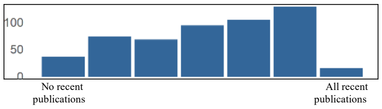	N=506<br>Admitted=472<br>Weighted mean =3.4	SD=3.2<br>Proportion (no/yes) =0.07, 0.93<br>POMP=0.57
22	HARKing in confirmatory qualitative studies	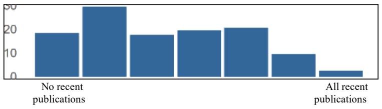	N=114<br>Admitted=96<br>Weighted mean =2.7	SD=2.6<br>Proportion (no/yes) =0.16, 0.84<br>POMP=0.45
23	Do not distinguish between statistical and practical significance	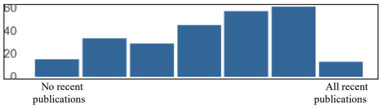	N=246<br>Admitted=232<br>Weighted mean =3.5	SD=3.3<br>Proportion (no/yes) =0.06, 0.94<br>POMP=0.59
24	Report non-significant findings as evidence for no effect	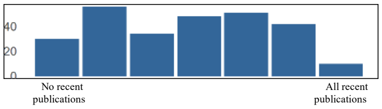	N=264<br>Admitted=235<br>Weighted mean =3.1	SD=2.9<br>Proportion (no/yes) =0.11, 0.89<br>POMP=0.51
25	Plagiarizing other researchers' unpublished ideas	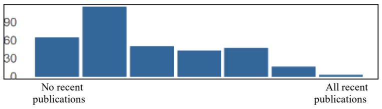	N=327<br>Admitted=264<br>Weighted mean =2.2	SD=2.1<br>Proportion (no/yes) =0.19, 0.81<br>POMP=0.37

*N = number of statements; Admitted = non-zero answers; weighted mean = mean of non-zero answers (1-6); sd = standard deviation of weighted mean; proportion = zero and non-zero answers; POMP = Converting scores to the Percent Of Maximum Possible score [(observed score - minimum score on the scale)/(maximum score on the scale - minimum score on the scale)].

S22 Table. Questionable Research Practices (QRPs): Self-reported use for the Danish respondents

No.	QRP	Distribution	Descriptive statistics	
1	Honorary authorships	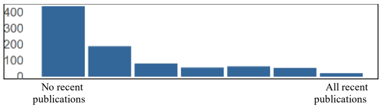	N=841<br>Admitted=412<br>Weighted mean =2.4	SD=2.4<br>Proportion (no/yes) =0.51, 0.49<br>POMP=0.4
2	Fail to offer deserved authorship to collaborators	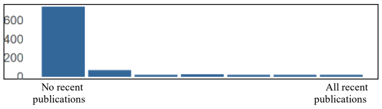	N=821<br>Admitted=89<br>Weighted mean =2.1	SD=2.2<br>Proportion (no/yes) =0.89, 0.11<br>POMP=0.35
3	Not disclosing relevant conflicts of interests	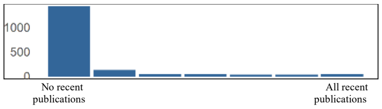	N=1,589<br>Admitted=176<br>Weighted mean =2.3	SD=2.4<br>Proportion (no/yes) =0.89, 0.11<br>POMP=0.38
4	Collect more data if results are non-significant	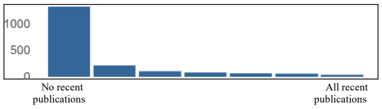	N=1,698<br>Admitted=397<br>Weighted mean =2.2	SD=2.2<br>Proportion (no/yes) =0.77, 0.23<br>POMP=0.37
5	Undisclosed data dredging, p-hacking	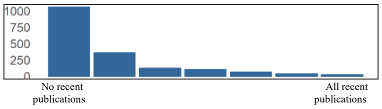	N=1,710<br>Admitted=656<br>Weighted mean =2	SD=1.9<br>Proportion (no/yes) =0.62, 0.38<br>POMP=0.33
6	Deliberately publishing redundant work	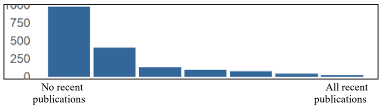	N=1,608<br>Admitted=650<br>Weighted mean =1.8	SD=1.7<br>Proportion (no/yes) =0.60, 0.40<br>POMP=0.3
7	Cite literature without reading read it	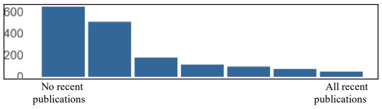	N=1573<br>Admitted=934<br>Weighted mean =2.1	SD=2.1<br>Proportion (no/yes) =0.41, 0.59<br>POMP=0.34
8	Claim to have used a qualitative approach appropriately, when this was not the case	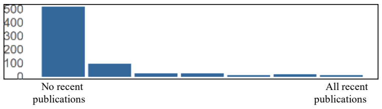	N=637<br>Admitted=129<br>Weighted mean =1.8	SD=1.7<br>Proportion (no/yes) =0.80, 0.20<br>POMP=0.29
9	Avoid to share data, code, protocol etc. requested by colleagues	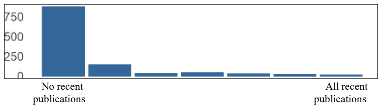	N=1,104<br>Admitted=239<br>Weighted mean =2	SD=2<br>Proportion (no/yes) =0.78, 0.22<br>POMP=0.34
10	Agree to review a manuscript knowing that you have inadequate expertise	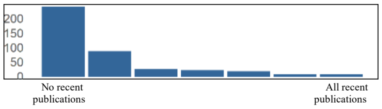	N=382<br>Admitted=144<br>Weighted mean =1.9	SD=1.8<br>Proportion (no/yes) =0.62, 0.38<br>POMP=0.32
11	Lack of sufficient effort when reviewing	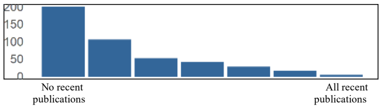	N=427<br>Admitted=230<br>Weighted mean =2.1	SD=2<br>Proportion (no/yes) =0.46, 0.54<br>POMP=0.36
12	Submitted a biased review report that evaluated the manuscript unfairly	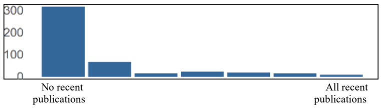	N=420<br>Admitted=109<br>Weighted mean =2.1	SD=2.2<br>Proportion (no/yes) =0.74, 0.26<br>POMP=0.36

13	Reuse previously published data without disclosure	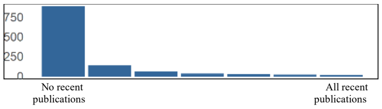	N=1,085<br>Admitted=211<br>Weighted mean =1.8	SD=1.7<br>Proportion (no/yes) =0.81, 0.19<br>POMP=0.31
14	Salami-slicing publications	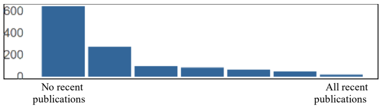	N=1,135<br>Admitted=502<br>Weighted mean =2.1	SD=2<br>Proportion (no/yes) =0.56, 0.44<br>POMP=0.34
15	Cite irrelevant literature to please	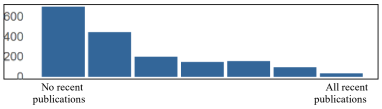	N=1,688<br>Admitted=1,001<br>Weighted mean =2.3	SD=2.3<br>Proportion (no/yes) =0.41, 0.59<br>POMP=0.39
16	Selective over-citing of own publications	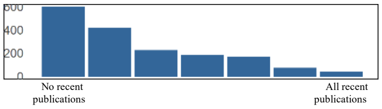	N=1,649<br>Admitted=1,057<br>Weighted mean =2.4	SD=2.3<br>Proportion (no/yes) =0.36, 0.64<br>POMP=0.4
17	Disregard citing relevant contradictory works	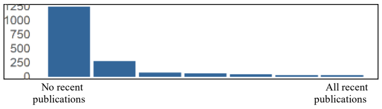	N=1,594<br>Admitted=372<br>Weighted mean =1.6	SD=1.5<br>Proportion (no/yes) =0.77, 0.23<br>POMP=0.27
18	Cherry-pick what supports a hypotheses and disregard that which does not	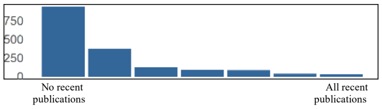	N=1,563<br>Admitted=639<br>Weighted mean =1.9	SD=1.9<br>Proportion (no/yes) =0.59, 0.41<br>POMP=0.32
19	Refrain from reporting findings that could weaken or contradict your findings	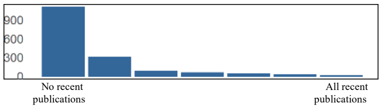	N=1,572<br>Admitted=474<br>Weighted mean =1.8	SD=1.6<br>Proportion (no/yes) =0.70, 0.30<br>POMP=0.29
20	Overselling results	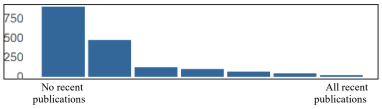	N=1,604<br>Admitted=718<br>Weighted mean =1.7	SD=1.6<br>Proportion (no/yes) =0.55, 0.45<br>POMP=0.29
21	HARKing in confirmatory quantitative studies	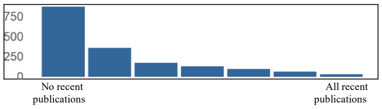	N=1,614<br>Admitted=755<br>Weighted mean =2.2	SD=2.1<br>Proportion (no/yes) =0.53, 0.47<br>POMP=0.36
22	HARKing in confirmatory qualitative studies	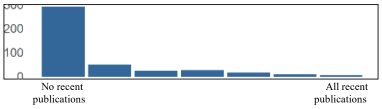	N=386<br>Admitted=99<br>Weighted mean =2.1	SD=2<br>Proportion (no/yes) =0.74, 0.26<br>POMP=0.35
23	Do not distinguish between statistical and practical significance	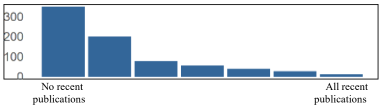	N=720<br>Admitted=375<br>Weighted mean =2	SD=2<br>Proportion (no/yes) =0.48, 0.52<br>POMP=0.34
24	Report non-significant findings as evidence for no effect	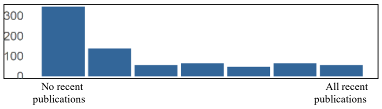	N=722<br>Admitted=382<br>Weighted mean =3	SD=3<br>Proportion (no/yes) =0.47, 0.53<br>POMP=0.49
25	Plagiarizing other researchers' unpublished ideas	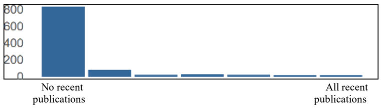	N=910<br>Admitted=91<br>Weighted mean =1.6	SD=1.6<br>Proportion (no/yes) =0.90, 0.10<br>POMP=0.27

*N = number of statements; Admitted = non-zero answers; weighted mean = mean of non-zero answers (1-6); sd = standard deviation of weighted mean; proportion = zero and non-zero answers; POMP = Converting scores to the Percent Of Maximum Possible score [(observed score - minimum score on the scale)/(maximum score on the scale - minimum score on the scale)].

S23 Table. Questionable Research Practices (QRPs): Self-reported use for the international respondents

No.	QRP	Distribution	Descriptive statistics	
1	Honorary authorships	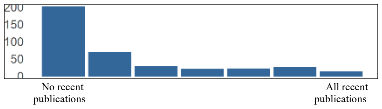	N=364<br>Admitted=165<br>Weighted mean=2.6	SD=2.7<br>Proportion (no/yes) =0.55, 0.45<br>POMP=0.44
2	Fail to offer deserved authorship to collaborators	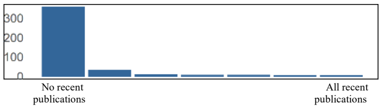	N=400<br>Admitted=46<br>Weighted mean=1.8	SD=1.7<br>Proportion (no/yes) =0.89, 0.12<br>POMP=0.29
3	Not disclosing relevant conflicts of interests	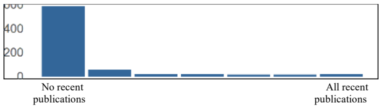	N=656<br>Admitted=81<br>Weighted mean=2.2	SD=2.4<br>Proportion (no/yes) =0.88, 0.12<br>POMP=0.37
4	Collect more data if results are non-significant	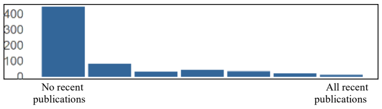	N=618<br>Admitted=180<br>Weighted mean=2.4	SD=2.3<br>Proportion (no/yes) =0.71, 0.29<br>POMP=0.4
5	Undisclosed data dredging, p-hacking	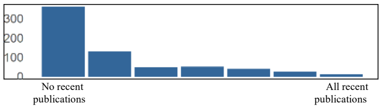	N=626<br>Admitted=269<br>Weighted mean=2.2	SD=2.2<br>Proportion (no/yes) =0.57, 0.43<br>POMP=0.37
6	Deliberately publishing redundant work	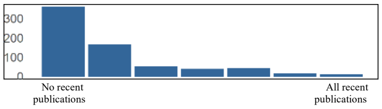	N=651<br>Admitted=294<br>Weighted mean=2	SD=1.9<br>Proportion (no/yes) =0.55, 0.45<br>POMP=0.33
7	Cite literature without reading read it	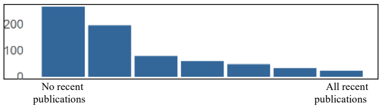	N=663<br>Admitted=401<br>Weighted mean=2.2	SD=2.2<br>Proportion (no/yes) =0.40, 0.60<br>POMP=0.37
8	Claim to have used a qualitative approach appropriately, when this was not the case	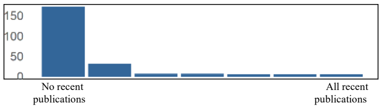	N=214<br>Admitted=44<br>Weighted mean=2	SD=2.1<br>Proportion (no/yes) =0.79, 0.21<br>POMP=0.33
9	Avoid to share data, code, protocol etc. requested by colleagues	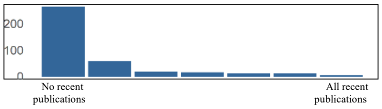	N=356<br>Admitted=95<br>Weighted mean=2	SD=1.9<br>Proportion (no/yes) =0.73, 0.27<br>POMP=0.33
10	Agree to review a manuscript knowing that you have inadequate expertise	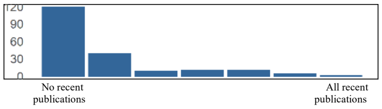	N=186<br>Admitted=68<br>Weighted mean=2	SD=1.9<br>Proportion (no/yes) =0.63, 0.37<br>POMP=0.33
11	Lack of sufficient effort when reviewing	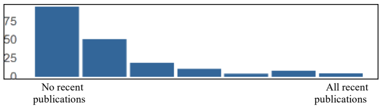	N=179<br>Admitted=86<br>Weighted mean=1.9	SD=1.9<br>Proportion (no/yes) =0.52, 0.48<br>POMP=0.32
12	Submitted a biased review report that evaluated the manuscript unfairly	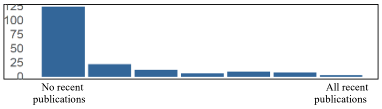	N=167<br>Admitted=45<br>Weighted mean=2.2	SD=2.2<br>Proportion (no/yes) =0.73, 0.27<br>POMP=0.37

13	Reuse previously published data without disclosure	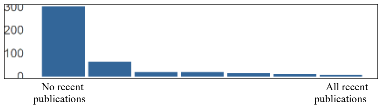	N=396<br>Admitted=99<br>Weighted mean=1.9	SD=1.8<br>Proportion (no/yes) =0.75, 0.25<br>POMP=0.32
14	Salami-slicing publications	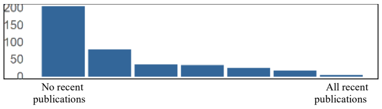	N=373<br>Admitted=172<br>Weighted mean=2.2	SD=2.1<br>Proportion (no/yes) =0.54, 0.46<br>POMP=0.37
15	Cite irrelevant literature to please	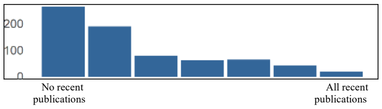	N=688<br>Admitted=427<br>Weighted mean=2.4	SD=2.3<br>Proportion (no/yes) =0.38, 0.62<br>POMP=0.39
16	Selective over-citing of own publications	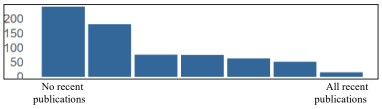	N=674<br>Admitted=436<br>Weighted mean=2.4	SD=2.4<br>Proportion (no/yes) =0.35, 0.65<br>POMP=0.41
17	Disregard citing relevant contradictory works	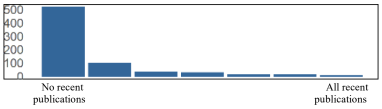	N=681<br>Admitted=166<br>Weighted mean=1.9	SD=1.8<br>Proportion (no/yes) =0.76, 0.24<br>POMP=0.31
18	Cherry-pick what supports a hypotheses and disregard that which does not	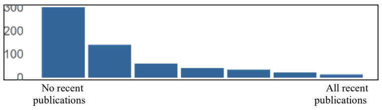	N=567<br>Admitted=271<br>Weighted mean=2.1	SD=2<br>Proportion (no/yes) =0.52, 0.48<br>POMP=0.35
19	Refrain from reporting findings that could weaken or contradict your findings	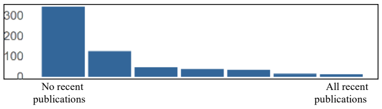	N=569<br>Admitted=231<br>Weighted mean=2.1	SD=2<br>Proportion (no/yes) =0.59, 0.41<br>POMP=0.34
20	Overselling results	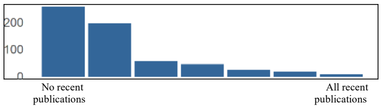	N=575<br>Admitted=321<br>Weighted mean=1.8	SD=1.7<br>Proportion (no/yes) =0.44, 0.56<br>POMP=0.3
21	HARKing in confirmatory quantitative studies	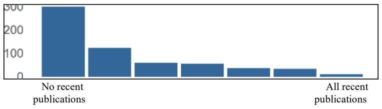	N=580<br>Admitted=286<br>Weighted mean=2.3	SD=2.3<br>Proportion (no/yes) =0.51, 0.49<br>POMP=0.39
22	HARKing in confirmatory qualitative studies	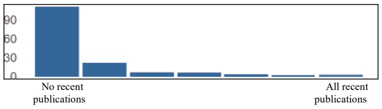	N=141<br>Admitted=32<br>Weighted mean=1.8	SD=1.7<br>Proportion (no/yes) =0.77, 0.23<br>POMP=0.29
23	Do not distinguish between statistical and practical significance	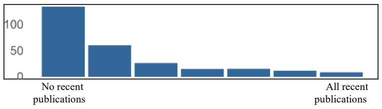	N=253<br>Admitted=121<br>Weighted mean=2.3	SD=2.3<br>Proportion (no/yes) =0.52, 0.48<br>POMP=0.38
24	Report non-significant findings as evidence for no effect	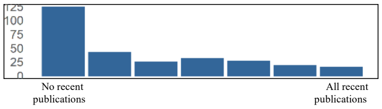	N=274<br>Admitted=151<br>Weighted mean=3	SD=2.9<br>Proportion (no/yes) =0.45, 0.55<br>POMP=0.49
25	Plagiarizing other researchers' unpublished ideas	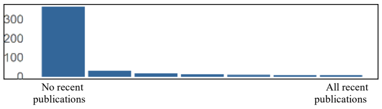	N=405<br>Admitted=44<br>Weighted mean=1.8	SD=1.7<br>Proportion (no/yes) =0.89, 0.11<br>POMP=0.3

*N = number of statements; Admitted = non-zero answers; weighted mean = mean of non-zero answers (1-6); sd = standard deviation of weighted mean; proportion = zero and non-zero answers; POMP = Converting scores to the Percent Of Maximum Possible score [(observed score - minimum score on the scale)/(maximum score on the scale - minimum score on the scale)].

S24 Figure. Predictors of self-reported prevalence. Models where Danish and international surveys are combined.

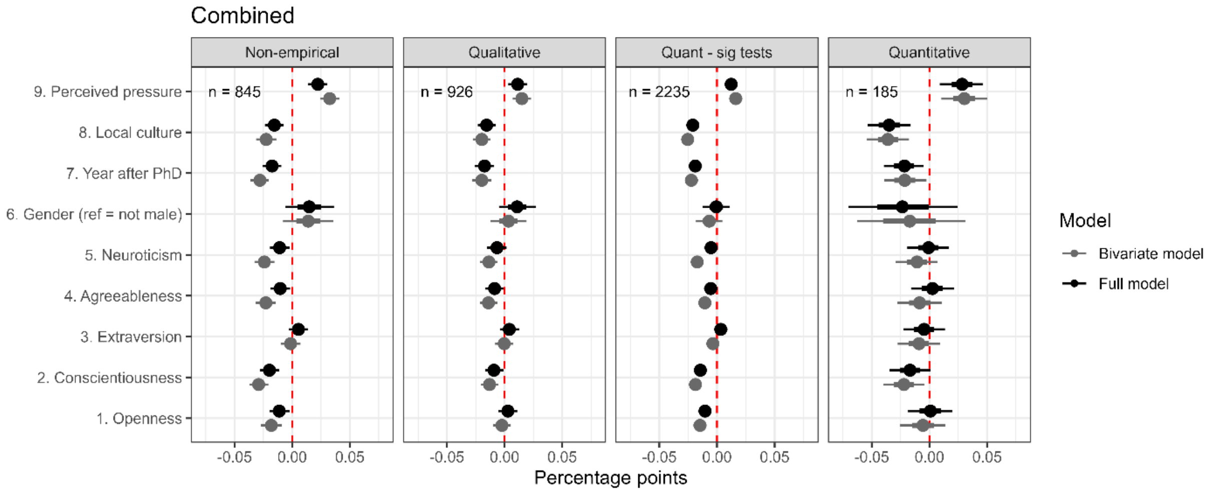

S24 Fig. Predictors of self-reported prevalence: Danish and survey combined.
Response patterns across the four research approaches. Bayesian Linear Probability Model reported with medians and quantile intervals 66-95% Credible Intervals.

S25 Figure. Predictors of self-reported prevalence. Models where Danish and international surveys are combined.

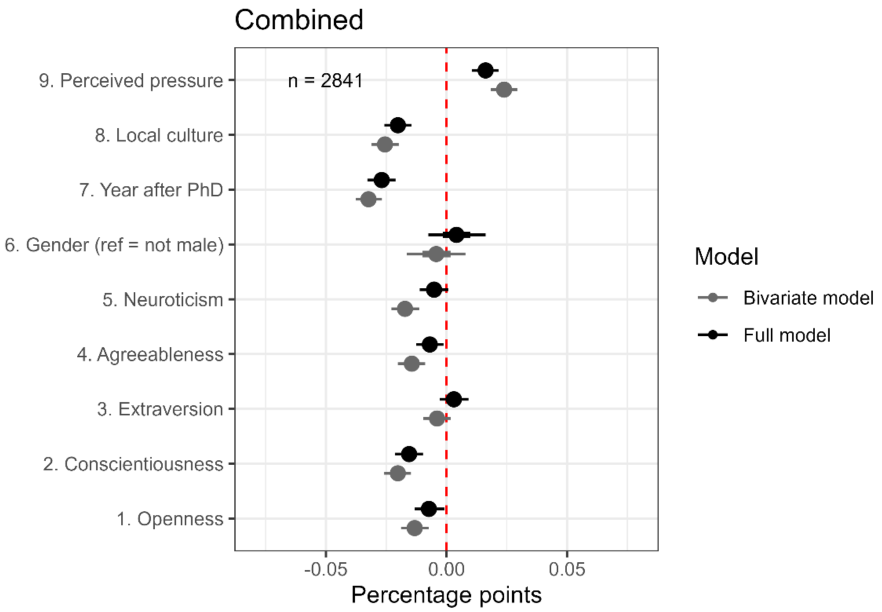

S25 Fig. Predictors of self-reported prevalence: Danish and survey combined.
Comparison of the Danish and international response patterns based on a subset of 11 QRP statements eligible to all respondents. Bayesian Linear Probability Model reported with medians and quantile intervals 66-95% Credible Intervals.

References

1. Wooldridge, J.M., *Introductory econometrics: A modern approach*. 6th ed. 2015: Cengage learning.
2. Clark, M. *Fractional Regression*. Retrieved from https://m-clark.github.io/posts/2019-08-20-fractional-regression/ 2019, Aug. 20 [cited December 16, 2021.
