# Supplementary material for: Is something rotten in the state of Denmark? Cross-national evidence for widespread involvement but not systematic use of questionable research practices across all fields of research
Source: PLoS One. 2024 Aug 12;19(8):e0304342. doi: 10.1371/journal.pone.0304342 (PMC11318862; doi:10.1371/journal.pone.0304342)
Supplement: S3 File — (PDF) [file pone.0304342.s004.pdf]

S25 Table. Estimates of self-reported use in comparable surveys.

		Present surveys		Borad set of QRPs		Narrow set of QRPs using statements from John et al. (2012)								Narrow set of QRPs using statements from John et al. (2012) plus three more			Special studies [1]meta-review [2]multiple-items		
QRR no.	Labels used in FIG #	Danish	International	Artino et al. (2019) [12]	Necker et al. (2014) [50]	John et al. (2012) [65a]	John et al. (2012) [65b]	Fiedler et al. (2016) [63]	Agnoli et al. (2017) [62]	Fraser et al. (2018) [13a]	Fraser et al. (2018) [13b]	Rabelo et al. (2020) [68]	Latan et al. (2021) [71]	Bakker et al. (2020) [69]	Makel et al. (2021) [67]	Chin et al. (2021) [70]	Xie et al. (2021) [60]	Flanagin et al. (1998) [88]	Rajasekaran et al. (2014) [89]
1	Honorary authorships	49	45	61 (32)													16[1]	19[2]	55[2]
2	Fail to offer deserved authorship to collaborators	11	12	6 (33)	1.5 (36)														
3	Not disclosing relevant conflicts of interests	11	12	3 (25)<br>3 (26)															
4	Collect more data if results are non-significant	23	29	26 (17)		56 (2)	58 (2)	33 (2)	53 (2)	37 (2)	51 (2)	22 (2)	59 (2)	23 (2)	29 (2)	15 (2)			
5	Undisclosed data dredging, p-hacking	38	43	12 (14)<br>20 (15)	4 (34)<br>7 (35)<br>37 (40)<br>38 (41)	22 (5)<br>23 (7)	38 (5)<br>43 (7)	22 (5)<br>40 (7)	22 (5)<br>40 (7)	27 (5)<br>24 (7)	18 (5)<br>24 (7)	18 (5)<br>20 (7)	23 (5)<br>43 (7)	24 (5)<br>34 (7)<br>46 (11)<br>45 (12)	29 (5)<br>25 (7)<br>42 (11)<br>50 (12)	27 (5)<br>24 (7)<br>32 (11)<br>39 (12)			
7	Cite literature without reading read it	59	60	50 (29)	52 (38)														
8	Claim to have used a qualitative approach appropriately, when this was not the case	20	21	24 (21)<br>23 (22)															
9	Avoid to share data, code, protocol etc. requested by colleagues	22	27	5 (31)															
14	Salami-slicing publications	44	46	22 (23)	20 (43)														
15	Cite irrelevant literature to please	59	62	50 (28)	59 (42)														
16	Selective over-citing of own publications	64	65	30 (30)															
17	Disregard citing relevant contradictory works	23	24		21 (37)														
18	Cherry-pick what supports a hypotheses and disregard that which does not	41	48	10 (18)															
19	Refrain from reporting findings that could weaken or contradict your findings	30	41	8 (20)	32 (39)	63 (1)<br>46 (6)	67 (1)<br>50 (6)	34 (1)<br>42 (6)	48 (1)<br>40 (6)	64 (1)	64 (1)	22 (1)<br>55 (6)	58 (1)<br>49 (6)	60 (6)<br>64 (10)	62 (6)<br>67 (10)	43 (6)<br>53 (10)			
20	Overselling results	45	56	28 (16)<br>9 (24)															
21	HARKing in confirmatory quantitative studies	47	49	26 (19)		27 (8)	35 (8)	47 (8)	37 (8)	49 (8)	54 (8)	9 (8)	37 (8)	46 (8)	46 (8)	29 (8)			
25	Plagiarizing other researchers' unpublished ideas	10	11	5 (27)															

NOTES: The estimates are the percentage of respondents saying they used the practice at least once in recent publications. In parentheses (..) are a reference number for the actual QRP statement in the comparable survey. Same numbers indicate similar statements in the comparable surveys. S#-# Tables provide a concordance between the reference number in the table (indicated with $), to the statements used in the comparable survey and the statement used in the present questionnaire.

S26 Table. Statements from comparable survey studies with a narrow set of QRPs using statements from John et al. (2012).

**Reference No.**	**Abbreviations** (from #70 Chin et al., 2021)	**Survey statements** First nine are from #65 John et al. (2012). If not included the study is mentioned	**Comparable to QRP in present surveys** (category)	**QRP statements in present surveys**	**Abbreviated QRP statements used in figures 2-4**
$1	Underreport outcomes	Failing to report all dependent measures that are relevant to a finding (Not in #70: Chin et al., 2021; (#69: Bakker et al., 2020; #67 Makel et al., 2021)	#19: Selective reporting	Deliberately refrain from reporting findings that could weaken or contradict own theories, hypotheses or findings.	Deliberately refrain from reporting findings that could weaken or contradict own theories, hypotheses, or findings.
$2	Sample selectively (1)	Collecting more data after seeing whether results are significant in order to render non-significant results significant	#4: Selective analysis	In significance testing studies, continue to collect more data in order to render non-significant results significant. For example: If initial results are not statistically significant, collect more data until you get the desired result.	Collect more data if results are non-significant
$3	Underreport conditions	Failing to report all conditions that are relevant to a finding	#19: Selective reporting	Deliberately refrain from reporting findings that could weaken or contradict own theories, hypotheses or findings.	Deliberately refrain from reporting findings that could weaken or contradict own theories, hypotheses, or findings.
4	Sample selectivity (2)	Stopping data collection after achieving the desired result concerning a specific finding	No comparable QRP statements		
$5	Round p-values	Rounding off p values (e.g., reporting a p-value of .054 as .05)	#5: Selective analysis	In significance testing studies, continue to reanalyse data until a statistically significant result is obtained. For example: If initial results are not statistically significant, remove or transform data; use other statistical tests; redefine the outcome variable, switching to an alternate control group; trying various combinations of independent and control variables; analysing various subgroups and so forth; thus, reanalysing data until you get the desired result.	Undisclosed data dredging, p-hacking
$6	Omit non-significant studies or variables	Selectively reporting results regarding a specific finding that 'worked' (Not in #13: Fraser et al., 2018)	#19: Selective reporting	Deliberately refrain from reporting findings that could weaken or contradict own theories, hypotheses or findings.	Deliberately refrain from reporting findings that could weaken or contradict own theories, hypotheses, or findings.
$7	Exclude data selectively	Deciding whether to exclude data after looking at the impact of doing so regarding a specific finding	#5: Selective analysis	In significance testing studies, continue to reanalyse data until a statistically significant result is obtained. For example: If initial results are not statistically significant, remove or transform data; use other statistical tests; redefine the	Undisclosed data dredging, p-hacking

				outcome variable, switching to an alternate control group; trying various combinations of independent and control variables; analysing various subgroups and so forth; thus, reanalysing data until you get the desired result.	
$8	HARKing	Claiming to have predicted an unexpected result	#21: Selective analysis	Without disclosure, formulate or change hypotheses after having seen the results, thereby presenting an unexpected finding as having been predicted from the start in the form of a research hypothesis (quantitative study).	HARKing in confirmatory quantitative studies
9	Mislead about demographic effects	Claiming that results are unaffected by demographic variables (e.g., gender) despite being unsure (or knowing that they are)	No comparable QRP statements		

S27 Table. Statements from comparable survey studies with a narrow set of QRPs using statements from John et al. (2012) plus three more statements.

**Reference No.**	**Abbreviations** (in studies)	**Survey statements** These three are only in #70: Chin et al. (2021); #69: Bakker et al. (2020); #67 Makel et al.(2021)	**Comparable to QRP in present surveys** (category)	**QRP statements in present surveys**	**Abbreviated QRP statements used in figures 2-4**
$10	Underreport results<br><br>Not reporting full analyses<br><br>Omitting analyses	Reporting a set of results as the complete set of analyses when other analyses were also conducted (#70: Chin et al., 2021)<br><br>Reporting a set of results as the complete set of analyses when other analyses were also conducted but these are not reported. (#69: Bakker et al., 2020)<br><br>Reporting a set of results as the complete set of analyses when other analyses were also conducted. (#67 Makel et al., 2021)	#19: Selective reporting	Deliberately refrain from reporting findings that could weaken or contradict own theories, hypotheses or findings.	Deliberately refrain from reporting findings that could weaken or contradict own theories, hypotheses, or findings.
$11	Drop covariates selectively<br><br>Adding or dropping variables	Not reporting covariates that failed to reach statistical significance (e.g. p < 0.05) or some other desired statistical threshold. (#70: Chin et al., 2021)<br><br>Adding or dropping covariates in order to	#5: Selective analysis	In significance testing studies, continue to reanalyse data until a statistically significant result is obtained. For example: If initial results are not statistically significant, remove or transform data; use other statistical tests; redefine the outcome variable, switching	Undisclosed data dredging, p-hacking

	Omitting non-significant covariates	reach statistical significance (e.g., $p < .05$) on a key variable.<br>(#69: Bakker et al., 2020)<br><br>Not reporting covariates that failed to reach statistical significance (e.g., $p \leq .05$) or some other desired statistical threshold<br>(#67 Makel et al., 2021)		to an alternate control group; trying various combinations of independent and control variables; analysing various subgroups and so forth; thus, reanalysing data until you get the desired result.	
S12	Switch analysis selectively<br><br>Changing statistical test<br><br>Analysis gaming	Changing to another type of statistical analysis after the analysis initially chosen failed to reach statistical significance (e.g. $p < 0.05$) or some other desired statistical threshold<br>(#70: Chin et al., 2021)<br><br>Adopting another type of statistical analysis after the analysis initially chosen failed to reach statistical significance. For instance, using OLS instead of logit.<br>(#69: Bakker et al., 2020)<br><br>Changing to another type of statistical analysis after the analysis initially chosen failed to reach statistical significance (e.g., $p \leq .05$) or some other desired statistical threshold<br>(#67 Makel et al., 2021)	#5: Selective analysis	In significance testing studies, continue to reanalyse data until a statistically significant result is obtained. For example: If initial results are not statistically significant, remove or transform data; use other statistical tests; redefine the outcome variable, switching to an alternate control group; trying various combinations of independent and control variables; analysing various subgroups and so forth; thus, reanalysing data until you get the desired result.	Undisclosed data dredging, p-hacking

S28 Table. Statements from the comparable survey study by Artino et al. (2019) with a broad list of QRPs.

No.	Abbreviations	Survey statements<br>#12 Artino et al. (2021)	Comparable to QRP in present surveys (category)	QRP statements in present surveys	Abbreviated QRP statements used in figures 2-4
S14		13. Reported a downwardly rounded P value (e.g., reporting that a P value of .054 is less than .05)	#5: Selective analysis	In significance testing studies, continue to reanalyse data until a statistically significant result is obtained. For example: If initial results are not statistically significant, remove or transform data; use other statistical tests; redefine the outcome variable, switching to an alternate control group; trying various combinations of independent and control variables; analysing various subgroups and so forth; thus, reanalysing data until you get the desired result.	Undisclosed data dredging, p-hacking
S15		15. Decided whether to exclude nonoutlier data after	#5: Selective analysis	In significance testing studies, continue to reanalyse data until	Undisclosed data dredging, p-hacking

		looking at the impact of doing so on the results		a statistically significant result is obtained. For example: If initial results are not statistically significant, remove or transform data; use other statistical tests; redefine the outcome variable, switching to an alternate control group; trying various combinations of independent and control variables; analysing various subgroups and so forth; thus, reanalysing data until you get the desired result.	
$16		16. In a qualitative study, failed to report disconfirming examples or cases that weaken your conclusions	#20: Spin	Wilfully present findings as more "clear-cut" than justified by the data. For example, neglecting to disclose contradictory results, or thoroughly discuss study limitations, or deliberately overlooking counter arguments.	Overselling results
$17		17. Collected more data after seeing that the results were almost statistically significant	#4: Selective analysis	In significance testing studies, continue to collect more data in order to render non-significant results significant. For example: If initial results are not statistically significant, collect more data until you get the desired result.	Collect more data if results are non-significant
$18		18. To confirm a hypothesis, selectively deleted or changed data after performing data analysis	#18: Selective reporting	Selectively focusing on parts of the data or source material that support your preconceptions or hypotheses and deliberately disregarding parts that do not. Source material can be of all kinds, such as interviewees, documents, primary sources, etc.	Cherry-pick what supports a hypotheses and disregard that which does not
$19		19. Reported an unexpected finding as having been hypothesized from the start	#21: Selective analysis	Without disclosure, formulate or change hypotheses after having seen the results, thereby presenting an unexpected finding as having been predicted from the start in the form of a research hypothesis (quantitative study).	HARKing in confirmatory quantitative studies
$20		20. Concealed results that contradicted your previous findings or convictions	#19: Selective reporting	Deliberately refrain from reporting findings that could weaken or contradict own theories, hypotheses or findings.	Deliberately refrain from reporting findings that could weaken or contradict own theories, hypotheses, or findings.
$21		21. Claimed you used a particular qualitative research approach appropriately (e.g., grounded theory) when you knowingly did not	#8: Misleading reporting	Claim to have used a particular qualitative analytical approach appropriately, for example "grounded theory" or "triangulation", when this was not the case.	Claim to have used a qualitative approach appropriately when this was not the case
$22		22. Claimed you used a particular qualitative research technique appropriately (e.g., saturation, triangulation) when you knowingly did not	#8: Misleading reporting	Claim to have used a particular qualitative analytical approach appropriately, for example "grounded theory" or "triangulation", when this was not the case.	Claim to have used a qualitative approach appropriately when this was not the case
$23		23. Spread study results over more papers than is appropriate ("salami slicing")	#14: Recycling	Deliberately divide the results of a study over more publications than needed with the intention to increase the number of publications.	Salami-slicing publications

S24		24. Deliberately failed to mention important limitations of a study in the published paper	#20: Spin	Wilfully present findings as more “clear-cut” than justified by the data. For example, neglecting to disclose contradictory results, or thoroughly discuss study limitations, or deliberately overlooking counter arguments.	Overselling results
S25		25. Deliberately failed to mention an organization that funded your research in the published paper	#3: Transparency	In a publication, failing to disclose relevant personal, financial, political or intellectual conflicts of interests.	Not disclosing relevant conflicts of interests
S26		28. Failed to disclose relevant financial or intellectual conflicts of interest	#3: Transparency	In a publication, failing to disclose relevant personal, financial, political or intellectual conflicts of interests.	Not disclosing relevant conflicts of interests
S27		29. Used someone else’s ideas without their permission or proper citation	#25: Plagiarism	Deliberately using another researcher’s unpublished idea without giving credit. For example, publishing an idea voiced by a colleague at an informal meeting without giving her/him credit.	Plagiarizing other researchers' unpublished ideas
S28		32. Selectively cited certain papers just to please editors or reviewers	#15: Citing practices	Selectively citing irrelevant or unnecessary publications to please reviewers or editors.	Cite irrelevant literature to please
S29		33. Cited articles and or materials that you have not read	#7: Citing practices	Citing literature deemed relevant for your study without actually having read it.	Cite literature without reading read it
S30		34. Selectively cited your own work just to improve your citation metrics	#16: Citing practices	Deliberately cite own publications more than warranted by their relevance, to promote the visibility of your work or improve your citation metrics.	Selective over-citing of own publications
S31		37. Refused to share data with legitimate colleagues	#9: Transparency	Avoiding to share data, research protocols, information on experimental setup, instrumentation, coding, or other information about a study requested by colleagues to evade transparency.	Avoid sharing data, code, protocol etc. requested by colleagues
S32		38. Added one or more authors to a paper who did not qualify for authorship (“honorary authorship”)	#1: Authorship	Including authors on a publication who has not contributed sufficiently to the work to merit authorship.	Honorary authorships
S33		41. Omitted a contributor who deserved authorship	#2: Authorship	Failing to offer authorship to collaborators who had contributed sufficiently to the work to merit authorship.	Fail to offer deserved authorship to collaborators

S29 Table. Statements from the comparable survey study by Necker (2012) with a broad list of QRPs.

**No.**	**Abbreviations**	**Survey statements** #50 Necker (2012)	**Comparable to QRP in present surveys** (category)	**QRP statements in present surveys**	**Abbreviated QRP statements used in figures 2-4**
S34		8. Excluded part of the data (e.g., outliers) without reporting this	#5: Selective analysis	In significance testing studies, continue to reanalyse data until a statistically significant result is obtained. For example: If initial results are not statistically significant, remove or transform data; use other	Undisclosed data dredging, p-hacking

				statistical tests; redefine the outcome variable, switching to an alternate control group; trying various combinations of independent and control variables; analysing various subgroups and so forth; thus, reanalysing data until you get the desired result.	
$35		9. Used tricks to increase t-value, R2, or other statistics	#5: Selective analysis	In significance testing studies, continue to reanalyse data until a statistically significant result is obtained. For example: If initial results are not statistically significant, remove or transform data; use other statistical tests; redefine the outcome variable, switching to an alternate control group; trying various combinations of independent and control variables; analysing various subgroups and so forth; thus, reanalysing data until you get the desired result.	Undisclosed data dredging, p-hacking
$36		10. Failed to correctly give a colleague co-authorship who has worked on the paper	#2: Authorship	Failing to offer authorship to collaborators who had contributed sufficiently to the work to merit authorship.	Fail to offer deserved authorship to collaborators
$37		11. Refrained from citing results or opinions that are not in line with your own analysis	#17: Citing practices	Deliberately disregard citing relevant publications that contradict own beliefs or research.	Disregard citing relevant contradictory works
$38		12. Refrained from checking the contents of the works cited	#7: Citing practices	Citing literature deemed relevant for your study without actually having read it.	Cite literature without reading read it
$39		14. Presented empirical findings selectively so that they confirm one's argument	#19: Selective reporting	Deliberately refrain from reporting findings that could weaken or contradict own theories, hypotheses or findings.	Deliberately refrain from reporting findings that could weaken or contradict own theories, hypotheses, or findings.
$40		15. Searched for control variables until you got the desired results	#5: Selective analysis	In significance testing studies, continue to reanalyse data until a statistically significant result is obtained. For example: If initial results are not statistically significant, remove or transform data; use other statistical tests; redefine the outcome variable, switching to an alternate control group; trying various combinations of independent and control variables; analysing various subgroups and so forth; thus, reanalysing data until you get the desired result.	Undisclosed data dredging, p-hacking
$41		16. Stopped statistical analysis when you had a desired result	#5: Selective analysis	In significance testing studies, continue to reanalyse data until a statistically significant result is obtained. For example: If initial results are not statistically significant, remove or transform data; use other statistical tests; redefine the outcome variable, switching to an alternate control group; trying various combinations of independent and control	Undisclosed data dredging, p-hacking

				variables; analysing various subgroups and so forth; thus, reanalysing data until you get the desired result.	
$42		19. Cited strategically to raise publication prospects (e.g., to please editors or possible referees)	#15: Citing practices	Selectively citing irrelevant or unnecessary publications to please reviewers or editors.	Cite irrelevant literature to please
$43		20. Maximized the number of publications by dividing the work to the smallest publishable unit, meaning several individual articles covering similar topics and differing from each other only slightly	#14: Recycling	Deliberately divide the results of a study over more publications than needed with the intention to increase the number of publications.	Salami-slicing publications

S30 Table. List of comparable surveys with specific information on domains examined, samples sizes and response rates, as well as practices comparable to the present questionnaire.

**Ref. no. in paper**	**Reference**	**Domains,** (no. of practices studied, broad set: yes/ no)	**Sample size** (% response rate)	**Practices comparable to this study**<br>n = actual sample if deviant from total<br>Estimates = percentage of self-reported use at least once
[12]	Artino Jr, A.R., E.W. Driessen, and L.A. Maggio, Ethical shades of grey: International frequency of scientific misconduct and questionable research practices in health professions education. Academic Medicine, 2019. 94(1): p. 76-84.	Health professions education (43, yes)	590 (22%)	$14 ≈ #5: data-dredging = 12% (n581)<br>$15 ≈ #5: data-dredging = 20% (n584)<br>$16 ≈ #20: overselling = 28% (n583)<br>$17 ≈ #4: sample selectivity = 26% (n583)<br>$18 ≈ #18: cherry-picking = 10% (n583)<br>$19 ≈ #21: HARKing = 26% (n583)<br>$20 ≈ #19: refrain from reporting = 8% (n583)<br>$21 ≈ #8: misleading reporting = 24% (n566)<br>$22 ≈ #8: misleading reporting = 23% (n566)<br>$23 ≈ #14: salami-slicing = 22% (n566)<br>$24 ≈ #20: overselling = 9% (n566)<br>$25 ≈ #3: conflicts of interest = 3% (n565)<br>$26 ≈ #3: conflicts of interest = 3% (n567)<br>$27 ≈ #25: plagiarism = 5% (n567)<br>$28 ≈ #15: cite to please = 50% (n566)<br>$29 ≈ #7: cite without reading = 50% (n565)<br>$30 ≈ #16: over self-citing = 30% (n562)<br>$31 ≈ #9: lack of sharing = 5% (n564)<br>$32 ≈ #1: honorary authorship = 61% (n563)<br>$33 ≈ #2: failing to offer authorship =6% (n563)
[13]	Fraser, H., et al., Questionable research practices in ecology and evolution. PLOS ONE, 2018. 13(7): p. e0200303.	Ecologist & evolutionary biologist (9, no)	494 & 313 (total 807 = 15%)	*[13a] Ecologist (n494):*<br>$1 ≈ #19: refrain from reporting = 64%<br>$2 ≈ #4: sample selectivity = 37%<br>$5 ≈ #5: data-dredging = 27%<br>$7 ≈ #5: data-dredging = 24%<br>$8 ≈ #21: HARKing =49%<br>Overall = 40%<br><br>*[13b] Evolutionary biologists (n313):*<br>$1 ≈ #19: refrain from reporting = 64%<br>$2 ≈ #4: sample selectivity = 51%<br>$5 ≈ #5: data-dredging = 18%<br>$7 ≈ #5: data-dredging = 24%<br>$8 ≈ #21: HARKing =54%<br>Overall = 42%
[50]	Necker, S., Scientific misbehavior in economics. Research Policy, 2014. 43(10): p. 1747-1759.	Economics (members of European Economics Association)	426 (17%)	$34 ≈ #5: data-dredging = 4% (n348)<br>$35 ≈ #5: data-dredging= 7% (n348)<br>$36 ≈ #2: failing to offer authorship = 1.5% (n423)<br>$37 ≈ #17: disregard relevant citing = 21% (n422)<br>$38 ≈ #7: cite without reading = 52% (n422)

		(18, yes)		$39 ≈ #19: refrain from reporting = 32% (n348)<br>$40 ≈ #5: data-dredging = 37% (n348)<br>$41 ≈ #5: data-dredging = 38% (n348)<br>$42 ≈ #15: cite to please = 59% (n420)<br>$43 ≈ #14: salami-slicing = 20% (n423)
[60]	Xie, Y., K. Wang, and Y. Kong, Prevalence of Research Misconduct and Questionable Research Practices: A Systematic Review and Meta-Analysis. Science and Engineering Ethics, 2021. 27(4): p. 41.	Meta-analyses of different studies from various different domains<br>(Many, yes)	42 articles included (571 studies extracted)	*Pooled estimates of various self-reported QRPs*<br>#1 honorary authorship = 16%<br>Overall (Unspecified QRPs) = 12.5%
[62]	Agnoli, F., et al., Questionable research practices among Italian research psychologists. PLOS ONE, 2017. 12(3): p. e0172792.	Members of Italian Psychology Association<br>(9, no)	277 (206 completed)<br>(24%)	$1 ≈ #19: refrain from reporting = 48% (n219)<br>$2 ≈ #4: sample selectivity = 53% (n222)<br>$5 ≈ #5: data-dredging = 22% (n221)<br>$6 ≈ #19: refrain from reporting = 40% (n217)<br>$7 ≈ #5: data-dredging = 40% (n219)<br>$8 ≈ #21: HARKing =37% (n219)<br>Overall = 30%
[63]	Fiedler, K. and N. Schwarz, Questionable Research Practices Revisited. Social Psychological and Personality Science, 2016. 7(1): p. 45-52.	Members of German Psychology Association<br>(9, no)	1,138<br>(35%)	$1 ≈ #19: refrain from reporting = 34%<br>$2 ≈ #4: sample selectivity = 33%<br>$5 ≈ #5: data-dredging = 22%<br>$6 ≈ #19: refrain from reporting = 42%<br>$7 ≈ #5: data-dredging = 40%<br>$8 ≈ #21: HARKing =47%<br>Overall = 28%
[65]	John, L.K., G. Loewenstein, and D. Prelec, Measuring the Prevalence of Questionable Research Practices With Incentives for Truth Telling. Psychological Science, 2012. 23(5): p. 524-532.	American psychologists<br>(9, no)	2,155 (36%)<br>1,436 (24%) completed responses	*[66a] Control group (n667; 466 completed):*<br>$1 ≈ #19: refrain from reporting = 63%<br>$2 ≈ #4: sample selectivity = 56%<br>$5 ≈ #5: data-dredging = 22%<br>$6 ≈ #19: refrain from reporting = 46%<br>$7 ≈ #5: data-dredging = 23%<br>$8 ≈ #21: HARKing =27%<br>Overall = 33%<br><br>*[66b] Incentives for truth telling group (n1,488; 970 completed):*<br>$1 ≈ #19: refrain from reporting = 67%<br>$2 ≈ #4: sample selectivity = 58%<br>$5 ≈ #5: data-dredging = 38%<br>$6 ≈ #19: refrain from reporting = 50%<br>$7 ≈ #5: data-dredging = 43%<br>$8 ≈ #21: HARKing =35%<br>Overall = 37%
[68]	Rabelo, A.L.A., et al., Questionable research practices among Brazilian psychological researchers: Results from a replication study and an international comparison. International Journal of Psychology, 2020. 55(4): p. 674-683.	Brazilian psychological researchers<br>(9, no)	232<br>(?%)	$1 ≈ #19: refrain from reporting = 22%<br>$2 ≈ #4: sample selectivity = 22%<br>$5 ≈ #5: data-dredging = 18%<br>$6 ≈ #19: refrain from reporting = 55%<br>$7 ≈ #5: data-dredging = 20%<br>$8 ≈ #21: HARKing =9%<br>Overall = 22%
[67]	Makel, M.C., et al., Both Questionable and Open Research Practices Are Prevalent in Education Research. Educational Researcher, 2021: p. 0013189X211001356.	Researchers publishing in American Educational Research Association journals<br>(10, no)	Different for each item, between 773 and 898<br>(~10%)	$2 ≈ #4: sample selectivity = 29% (n806)<br>$5 ≈ #5: data-dredging = 29% (n806)<br>$6 ≈ #19: refrain from reporting = 62% (n783)<br>$7 ≈ #5: data-dredging = 25% (n806)<br>$8 ≈ #21: HARKing =46% (n880)<br>$10 ≈ #19: refrain from reporting = 67% (n871)<br>$11 ≈ #5: data-dredging = 42% (n773)<br>$12 ≈ #5: data-dredging = 50% (n811)<br>Overall = 42%
[70]	Chin, J., et al., Questionable Research Practices and Open Science in	Researchers publishing in	1,612<br>(12%)	*Quantitative criminologists:*<br>$2 ≈ #4: sample selectivity = 15% (680)

	Quantitative Criminology. 2021, January 18, https://doi.org/10.31235/osf.io/bwm7s.	criminology or criminal justice journals indexed in Web of Science (10, no)		\$5 ≈ #5: data-dredging = 27% (670)<br>\$6 ≈ #19: refrain from reporting = 43% (n681)<br>\$7 ≈ #5: data-dredging = 24% (n679)<br>\$8 ≈ #21: HARKing =29% (n686)<br>\$10 ≈ #19: refrain from reporting = 53% (n677)<br>\$11 ≈ #5: data-dredging = 32% (n670)<br>\$12 ≈#5: data-dredging = 39% (n681)<br>Overall = 30%
[71]	Latan, H., et al., Crossing the Red Line? Empirical Evidence and Useful Recommendations on Questionable Research Practices among Business Scholars. Journal of Business Ethics, 2021.	Indonesian university business scholars	472 (39%)	\$1 ≈ #19: refrain from reporting = 58%<br>\$2 ≈ #4: sample selectivity = 59%<br>\$5 ≈ #5: data-dredging = 23%<br>\$6 ≈ #19: refrain from reporting = 49%<br>\$7 ≈ #5: data-dredging = 43%<br>\$8 ≈ #21: HARKing =37%<br>Overall = 40%
[69]	Bakker, B.N., et al., Questionable and open research practices: attitudes and perceptions among quantitative communication researchers. . 2020, November 18, https://doi.org/10.31234/osf.io/7uyn5.	Corresponding authors in communication research journals (9, no)	1,039 (28%)<br>872 (21%) completed	\$2 ≈ #4: sample selectivity = 23%<br>\$5 ≈ #5: data-dredging = 24%<br>\$6 ≈ #19: refrain from reporting = 60%<br>\$7 ≈ #5: data-dredging = 34%<br>\$8 ≈ #21: HARKing =46%<br>\$10 ≈ #19: refrain from reporting = 64%<br>\$11 ≈ #5: data-dredging = 46%<br>\$12 ≈#5: data-dredging = 45%<br>Overall = 43%
[88]	Flanagin, A., et al., Prevalence of Articles With Honorary Authors and Ghost Authors in Peer-Reviewed Medical Journals. JAMA, 1998. 280(3): p. 222-224.	Corresponding authors in 3 general medical journals (1, no)	809 (69%)	#1: honorary authorship ≈19%
[89]	Rajasekaran, S., R. Li Pi Shan, and J.T. Finnoff, Honorary Authorship: Frequency and Associated Factors in Physical Medicine and Rehabilitation Research Articles. Archives of Physical Medicine and Rehabilitation, 2014. 95(3): p. 418-428.	First authors of articles published in 3 major physical medicine and rehabilitation journals (1, no)	1,1182 (30%)	#1: honorary authorship ≈ 55%

NOTES: \$ indicates QRP statement in the comparable survey. # indicates the QRP number and statement in the present survey. S# Table provides a concordance where \$ can identified and the QRP statements can be compared. Overall means the mean self-reported use over all QRPs in the survey as reported in the actual surveys.
