# Supplementary material for: Is something rotten in the state of Denmark? Cross-national evidence for widespread involvement but not systematic use of questionable research practices across all fields of research
Source: PLoS One. 2024 Aug 12;19(8):e0304342. doi: 10.1371/journal.pone.0304342 (PMC11318862; doi:10.1371/journal.pone.0304342)
Supplement: Attachment — Submitted filename: response-to-reviewers.docx [file pone.0304342.s005.docx]

Response to Reviewers

# Journal requirements:

**[1]** When submitting your revision, we need you to address these additional requirements.

- Please ensure that your manuscript meets PLOS ONE's style requirements, including those for file naming.

**Response:**

- We have tried to ensure that the manuscript and files follow PLOS ONE’S style requirements.

**[2]** Thank you for stating the following financial disclosure:

[JWS 6183-00001B Danish Agency for Science and Higher Education (Ministry of Higher Education and Science) <https://ufm.dk/forskning-og-innovation/tilskud-til-forskning-og-innovation/hvem-har-modtaget-tilskud/2016/bevilling-til-forskning-i-dansk-forskningsintegritet-fra-styrelsen-for-forskning-og-innovation> No].

- Please state what role the funders took in the study. If the funders had no role, please state: “The funders had no role in study design, data collection and analysis, decision to publish, or preparation of the manuscript.”
- Please include this amended Role of Funder statement in your cover letter; we will change the online submission form on your behalf.

**Response:**

- We have added “The funders had no role in study design, data collection and analysis, decision to publish, or preparation of the manuscript”.
- We have included this amended Role of Funder statement in our revised cover letter.

**[3]** Thank you for stating the following in the Acknowledgments Section of your manuscript:

[This work is supported by the PRINT project (Practices, Perceptions, and Patterns of Research Integrity) funded by the Danish Agency for Science and Higher Education (Ministry of Higher Education and Science) under grant No 6183-00001B.

We thank Kaare Aagaard; Asger Dalsgaard Pedersen; Pernille Bak Pedersen (survey development), Emil B. Madsen (technical assistance), Allan Rye Lyngs (web scraping); Ana Marusic; Nicole Foeger (advise); Mads Sørensen, Tine Ravn (QRP development).]

We note that you have provided funding information that is not currently declared in your Funding Statement. However, funding information should not appear in the Acknowledgments section or other areas of your manuscript. We will only publish funding information present in the Funding Statement section of the online submission form.

Please remove any funding-related text from the manuscript and let us know how you would like to update your Funding Statement. Currently, your Funding Statement reads as follows:

[JWS 6183-00001B Danish Agency for Science and Higher Education (Ministry of Higher Education and Science)<https://ufm.dk/forskning-og-innovation/tilskud-til-forskning-og-innovation/hvem-har-modtaget-tilskud/2016/bevilling-til-forskning-i-dansk-forskningsintegritet-fra-styrelsen-for-forskning-og-innovation> No].

- Please include your amended statements within your cover letter; we will change the online submission form on your behalf.

**Response:**

- We have removed all funding-related text from the manuscript.
- We would like the Fundings Statement to be the following:

[This work is supported by the PRINT project (Practices, Perceptions, and Patterns of Research Integrity) funded by the Danish Agency for Science and Higher Education (Ministry of Higher Education and Science) under grant No JWS 6183-00001B]

- The Acknowledgment Section is changed to:

[We thank Kaare Aagaard; Asger Dalsgaard Pedersen; Pernille Bak Pedersen (survey development), Allan Rye Lyngs (web scraping); Ana Marusic; Nicole Foeger (advise); Mads Sørensen, Tine Ravn (QRP development).]

- Notice, we have removed “Emil B. Madsen (technical assistance)” from the acknowledgements and provided him with an authorship instead due to his strong involvement in revising the manuscript.

We have included the amended funder statement and change of authorship in our revised cover letter.

**[4]** We notice that your supplementary table S1 are included in the manuscript file.

- Please remove them and upload them with the file type 'Supporting Information'. Please ensure that each Supporting Information file has a legend listed in the manuscript after the references list.

**Response:**

- We have now upgraded Table S1 to the manuscript.

**[5]** Please include captions for your Supporting Information files at the end of your manuscript, and update any in-text citations to match accordingly.

- Please see our Supporting Information guidelines for more information: http://journals.plos.org/plosone/s/supporting-information.

**Response:**

- This has been done.

**[6]** Please include a copy of Table S2 in supporting information which you refer to in your text.

**Response:**

- This was a mistake. We have updated all supporting information according to the revisions suggested by Reviewer 1 and provide a new set of files listed in the Supporting Information.

# Responses to Reviewer 1

We would like to thank Reviewer 1 for relevant and incisive comments. We have attempted to address them to the best of our ability below.

**[1] Major: Using “QRP scores” that aggregate different QRP items for each respondent, resulting in invalid analyses and comparisons among groups.** The authors have an initial pool of 25 QRP items, all of which, presumably, have different true scores for use and prevalence in the population. However, these items are not all applicable to every researcher surveyed, as many pertain to practices that may not be used in a given field of research (e.g., historians will not p-hack, because p-values are probably not used in their field). To get around this problem, the study separated the respondents into 4 “preferred research approach” groups; according to the supplement, some items could be presented to every researcher (e.g., QRPs related to authorship or reviewing) and others only to a subset of researchers (e.g., QRPs related to significance testing only shown to researchers who reported their field uses significance testing). A total of 9 items were presented to each researcher, some of which appear to be mandatory depending on research approach and others were randomly sampled from a pool of eligible items, resulting in a measurement instrument for each participant that includes 9 of the 25 items. This in itself is not a problem — it actually solves two problems at once, by only presenting items that are more likely to be relevant to each participant, and reducing participant fatigue. But it has the downside of making aggregation of the responses extremely difficult or impossible: statements like “The median number of self-reported QRPs was three, and the average number 2.7 (DK) and 2.5 (INT). (p. 16)” become meaningless, because they are aggregated across different QRP statements. This would be similar to creating 25 math questions with a wide range of difficulties, then assigning different subsets across 4 classes, and testing each student using a semi-random subset of 9 questions. The average number of correct questions would hardly reflect anything meaningful about how good the students are at math, because they all took different tests with varying levels of difficulty. The tests would be different even within each classroom, so averages within or comparisons among these “preferred research approach” groups are invalid as well. Finally, comparisons across “main scientific fields”, depicted in Figure 2, are also problematic for similar reasons. Therefore, I urge the authors to remove most (or all) of the “aggregate results” section (pp. 16-17), as well as figures 1 and 2, from the manuscript, and not to use the aggregated score (or any form of aggregation of the responses to the 9 QRP statements presented to each individual) in any analyses. If the authors wish to make comparisons about the use or prevalence of QRPs with other studies in the literature, my suggestion is to select the items in this study that best match the items in the comparison study, and make careful item-by-item comparisons (e.g., “20% of respondents in this study admitted to having ever p-hacked, compared to 30% in John et al., and 22% in Smith et al.”). In my opinion, the results section should start with the self-reported use of the 25 individual QRPs, or research question 1 in the preregistration. If differences in QRPs for different “main scientific fields” are interesting enough to have their own figure, they could be displayed as separate estimates for each item and group, in a similar way as the results presented in the current figure 7.

**Response:**

- We thank Reviewer 1 for this pivotal methodological criticism. We agree that aggregating the 25 different QRPs in the way we have done may be problematic.
- In the revised manuscript we follow Reviewer 1’s suggestion and completely remove the “aggregate results” section including figures 1 and 2, as well as the aggregate results relating to “main scientific fields”.
- For the regression models predicting self-reported prevalence, we now base all models on analyses among respondents within the same “preferred research approaches” (see also comment 2b below). Here, Reviewer 1 also expresses some skepticism, but we do believe this is a much stronger design. Within the “preferred research approaches”, respondents here have been subjected to a much more consistent common pool of QRPs. This commonality allows for meaningful comparisons and analyses within each group, as the basis for comparison is uniform. Some statements are mandatory and others are randomly assigned within the constraints of each preferred approach, but – and this is key – within each preferred approach the random items are drawn from the *same* pool of items for *all* participants. Given that randomization is effective, then the effects of random assignment and missing items will cancel out at the aggregate level of each approach (this is essentially the logic of any missing-by-design questionnaire designs). This allows for a valid comparison of response patterns within each preferred approach group. By analyzing responses within each approach, we are inherently controlling for the variation in QRP exposure. This makes it easier to interpret differences or similarities in response patterns as being more directly related to the respondents’ perceptions and behaviors rather than the variability in which QRPs they were asked about.
- On this basis, the revised manuscript analyses data at the level of preferred research approaches as it accounts for the structured variability in QRP exposure. We therefore use this approach for modelling the predictors. At the same time, to accommodate the concerns of Reviewer 1 we reproduce our analyses only focusing on the set of 11 QRP items eligible to all participants. Reassuringly, this analysis replicates the main findings from analyses broken down into the four preferred research approaches.

**[2a] Major: The models used in the Predictors of Prevalence section use a “kitchen sink” approach, and an aggregated outcome.**

I need to preface this point by saying that I don’t know enough about Bayesian statistics to fully understand the models used in the “predictors of prevalence” section, or whether they are suitable for these analyses, so my review doesn't cover that. However, I’ll assume these are Bayesian versions of some form of multiple linear regression, or logistic regression. My specific concern is related to using all the predictors to model the outcome, regardless of how they may relate to each other. This approach makes assumptions about how the predictors are (or aren’t) related and ignores the (probably very complicated) relationships among these variables. The reported correlations on figures 6 and 7 are (or are akin to) partial correlations, or correlations “controlling” for all other predictors. This makes the interpretation of each coefficient very difficult, if not impossible, and can result in inappropriate estimates (e.g., see https://doi.org/10.1177/25152459221095823). This analysis would be clearer (and, I believe, much more informative) if, instead of this model with all the predictors, the authors presented a correlation matrix for all these variables. We could then see the actual correlation between, say, perceived pressure to publish and the use or QRPs without making assumptions about how those two variables are related to the other.

**Response:**

- We thank Reviewer1 for this observation.
- First, the Linear Probability Model (LPM) used in the manuscript is essentially a linear regression applied to bounded outcome data between 0 and 1. We have checked to what extent this model is useful by also applying fractional regression with a logit model to the data. The results are similar in which case the LPM seems more interpretable as it provides results on the outcome scale, whereas fractional regression reports log-odds which are harder to interpret.
- Second, we do not think our model can be designated as a “kitchen sink model”. The latter is typically used to imply that a long list of possible independent variables (IVs) is thrown into a model without much consideration for theoretical justification, relevance, risk of multicollinearity etc., in hope of finding some statistical pattern usually defined as IVs being statistically significant.
- As we argue in the manuscript, our models are exploratory, and we are not postulating any causal relations or make claims based on “statistical significance”. Nevertheless, it is certainly true that the interpretation of the model coefficients depends on the potential causal relationship between them, and that a full model with all predictors makes interpretation of each coefficient prone to “Table 2 fallacy”, where each is interpreted as a direct effect, even if the causal structure of the model lends itself to a myriad of indirect relationships. However, we do not think this is the case here. We specifically control for gender and academic age (years after PhD) to avoid their possible confounding effects, as engagement in QRPs, the OCEAN factors, and perceived pressure are known to vary according to both age and gender. In the revised figures 5-7, we also present the estimates for each predictor from a simple bivariate model (incl. only that predictor) for comparison with the estimates from the full model.
- We also argue that when the selection of variables is deliberate, and there’s a clear interest in examining the full model’s partial coefficients without engaging in a fishing expedition for statistical significance, this approach can be viewed as thorough rather than indiscriminate.
- Obviously, it can be discussed to what extent there is a theoretical justification for this, but as we argue this is a first attempt to bring these different sets of predictors together, not some kind of confirmatory causal analysis, and as such all covariates considered are plausible and chosen based on substantive grounds (i.e. previous research) for exploration in a descriptive and exploratory manner. We therefore think that our model is justified based on previous findings and suggestions.
- We agree with Reviewer 1 that our choice of predictors should be more clearly motivated. We have therefore revised this in the Analysis subsection on page 20 [manus with track-changes]:
  ““All predictors were carefully chosen. As already indicated, the systemic factors included have high predictive relevance since pressures are often cited as a cause for breaches in integrity [e.g., 30] and a supportive research culture is often pointed to as a plausible protective [e.g., 59]. Likewise, gender and academic age have also been examined before although their predictive value is unclear [e.g., 76]. What is novel in our approach is to combine these relevant systemic and demographic predictors with personality factors in the normal range. We conjecture that propensities for engagements in QRPs are a complicated mixture of experience, systemic and individual factors.”
- We also acknowledge that we should provide more information in relation to the limitations and challenges associated with including many predictors. As suggested by Reviewer 1, we have therefore provided a correlation matrix between all predictor variables, provided information about potential multicollinearity (none) and as stated above, also provided bivariate coefficients in the new plots in order to compare changes between a bivariate coefficient and its partial equivalent, i.e. when conditioning on the other covariates.

**[2b] Major: Using “QRP scores” that aggregate different QRP items for each respondent, resulting in invalid analyses and comparisons among groups.** Another issue with this analysis is that the outcome seems to be the same kind of aggregated “QRP usage” I mentioned in my previous point, and if it is, it should not be used as the outcome in this model, for the reasons described above. One alternative might be to report the correlations among all 9 predictors and their correlations with all 25 outcomes. That seems unwieldy at best but maybe there’s some way to present that without it becoming overwhelming. Maybe a correlation matrix among the predictors (to help readers understand which ones are correlated), and a figure similar to figs 3-5 showing the correlation of each QRP item with the 9 predictors? Or maybe a 9x25 heatmap (e.g., https://r-graph-gallery.com/heatmap) could be a good way to present these correlations between the 25 QRPs and the 9 predictors in a more visual way. I hope the authors find that at least one of these solutions works well.

**Response:**

- Again, we emphasize we are merely interested in response patterns, conditional associations in our data set between potential predictors and self-reported prevalence. Hence, we are interested in what extent these models provide a consistent picture of these predictors.
- As indicated above, our modelling analyses of predictors are now disaggregated into individual analyses for each of the four preferred research approaches, as well as a fifth analysis of all respondents but only based on the 11 QRPs which were eligible for all of them. The latter is used as a robustness check of the findings in the induvial approaches. As this subset implies fewer QRP statements per respondents, we excluded those respondents that only answered two or fewer of these potentially 11 statements.
- We present separate results for surveys and provide results for combined analyses where the two surveys are merged, in the supplementary material (S2 File).
- As suggested, we have also produced a correlation matrix in the supplementary material that shows correlation between predictors and QRPs.
- We also produced this explanation provided in the new Analyses subsection of Methods, page 19 [manus with track-changes]:
  “We present two main analyses. First, we conduct descriptive analyses to examine the distribution of responses to the 25 QRP statements from the two surveys, focusing on self-reported use, self-reported prevalence, and perceived prevalence (Figs 2-4). Subsequently, exploratory analyses were carried out to examine potential predictors of self-reported prevalence among the respondents. These analyses were performed on five subsets of respondents according to their preferred research approach. Since respondents were subjected to different subsets of the 25 QRPs depending on their choice of preferred research approach (see Fig 1), aggregate analyses based on all QRPs combined are not considered valid. Instead, analyses were conducted separately for each of the four preferred research approaches. Within these approaches, all respondents were, in principle, exposed to the same set of QRP statements, some statements were mandatory for the specific research approach, while others were randomly assigned from a common pool of 11 QRPs available to all respondents regardless of their preferred research approach. The latter also enables a fifth subset for analysis, namely all respondents in the surveys albeit on the restricted pool of 11 QRPs to which they all in principle have been randomly exposed.
  Given that randomization is effective, then the effects of random assignment and missing items will cancel out at the aggregate level of each subset examined. This allows for a valid comparison of response patterns within each of the five subsets. By analyzing responses within each subset, we were inherently controlling for the variation in QRP exposure. This makes it easier to interpret differences or similarities in response patterns as being more directly related to the respondents’ perceptions and behaviours, rather than the variability in which QRPs they were asked about.”
- Finally. We have re-written the entire Results subsection on “Predictors of prevalence” due to the disaggregate analyses, see [manus with track-changes].

**[3] Medium: Too many QRPs, not enough grouping**. The fact that a much wider diversity of practices is examined in this paper is a major strength, but it also makes it difficult to understand the results, especially when all 3 of the main figures (currently figs 3-5) display these practices in a different order. Both in the preregistration and in the paper, the authors suggest ways to group these practices — in the prereg, they mention 12 groups, and in the manuscript, they mention 9(?) groups (lines 5-7 of page 12, right above Table 2). Presenting figures 3-5 using (either of) those groupings, and with QRPs in the same order, would go a long way towards helping the reader digest the results, and would allow for comparison among the three figures.

**Response:**

- We acknowledge that we have been too inconsistent in the grouping of QRP statements. We have now settled for 11 categories and have used it consistently in the revised manuscript and supplementary material.
- However, we prefer to keep the presentation of results as they are in the figures previously numbered 3-5 (now 2-4) where QRPs are ranked according to frequency in the Danish survey. We find this presentation where QRPs are discussed and compared according to frequency in the Danish appropriate.
- Instead of grouping, we have added QRP numbers to the figure and in the text.

**[4] Medium: QRP statements**. The full list of QRP statements (and which groups of respondents got to see what subset of them) is only included in the supplement, despite being central to this study’s claims; at least some of this information should be included in the main manuscript. The authors can also concurrently introduce whatever grouping of these QRP statements they decide to use in addressing point #3 above.

- We agree that this should be more explicit in the manuscript. We have now promoted former S1 Table to the manuscript (Table 1). It provides abbreviations of the 25 QRP statements, their numbering, and categories. As mentioned above, categories are now fixed at 11.
- Further, we have provided a new figure (Fig 1) (see response to comment 7 below), that 1) shows the process of choosing the preferred research approach as suggested by Reviewer 1, and 2) shows which QRPs (indicated with numbers) are eligible to each of the four research approaches, as well as those which are eligible for all (indicated by an underscore).

**[5] Medium: Deviations from the preregistered analysis plan are not clearly indicated.** The preregistered analysis plan (https://osf.io/75g3d) includes a few dependent variables and analyses that are not in the paper and/or are only briefly mentioned, without explanation for why they were not included. I couldn’t find DV1 (participants’ “trust in research findings in their field of research”) and DV4 (participants’ “beliefs about how damaging such a QRP may be for the trust in research findings or claims in your specific field of research”) in the paper, there’s a list experiment that only gets a very brief mention in the limitations, and the explanation for why the peer review predictor wasn’t included in the paper isn't clear (more on that below). As they say, “preregistration is a plan, not a prison”; to me, it’s fine to make decisions that we couldn’t have foreseen before data collection, but which will make the paper better, especially with such a large dataset, but please make sure to report and explain all the deviations from the initial plan.

**Response:**

- We concur with Reviewer 1 that our initial attempt to address these deviations was insufficient and we appreciate the opportunity to provide a more comprehensive explanation here and in the revised manuscript.
- We must acknowledge that our preregistration of the project was in many ways imprudently conceived. Upon reflection and in light of the challenges we encountered, we tend to regret that we preregistered our project to the extent that we did. This regret stems not from a lack of commitment to research transparency and integrity, which we firmly uphold, but from a recognition that preregistration may not have been ideally suited to the inherently exploratory and fluid nature of our investigation.
- But as we did preregister our project, we should also be honest in reporting that, and as Reviewer 1 points out, also report when we deviate from it.
- Basically, our preregistration, while centered on the questionnaire, was originally thought of as encompassing our project in general, and therefore also numerous studies coming out of the survey, where the present manuscript is one of them. The nature of these studies, however, changed along the way and none were explicitly stated from the start. This has created some of the challenges and misalignments seemingly present in this manuscript.
- As we state in the opening of the preregistration, “our study is not confirmatory but will examine 3 main questions”. In essence, we now only address two of these questions in the present manuscript, and we deviate on at least five other issues.
- One. In this revised manuscript, we do not address use and prevalence across main fields of research as suggested in question 1, due to the changes in the aggregate analyses suggested by Reviewer 1 in the first comment.
- Two. We have clarified the issue with categories and settled at 11, see comment 3.
- Three. In the preregistration, we list four “key” dependent variables. Two of them, as Reviewer 1 points out, are not included in this manuscript (i.e. this study). These are DV1 and DV4 which both address “trust”. The reason we have not included these is scope. We simply do not have space for them in this manuscript. Obviously, we should have recognized that to begin with and stipulated more clearly in the preregistration that not everything would go into the same all-encompassing study.
- Four. We also preregistered a list experiment (LE) which was implemented in the questionnaire to try to examine potential social desirability bias. The results of the list experiment did not perform as expected. As we indicated in the manuscript by reference to [103] others have seemingly experienced similar issues with LE in comparable surveys and question their effectiveness and usefulness. But we also encountered technical issues with the LE implementation that we cannot account for, therefore we chose to disregard the LE as it did not work as intended. A final and recent concern is that the LE is based on the aggregate set of responses which have been removed from this revised manuscript as a valid level of analysis as suggested by Reviewer 1.
- Five. We mentioned that we would explore different kinds of regression models, but we ended up reporting one that we did not mention. The reason is that this model is easier to interpret and it provides similar results as those initially suggested.
- Six. Finally, we stated that we would use “factor analysis (EFA)… to correlate responses to questions concerning research contexts and conditions such as publication and peer review practices and local reward structures”. As Reviewer 1 rightly points out what we labelled the “peer review predictor” was discarded. We agree that our explanation for this was wanting. In the manuscript we argued that the EFA indicated that only two constructs were viable from the batteries examining research conditions: ‘perceived pressure’ and ‘local research culture’. While in our view, this interpretation still holds up, it is irrelevant as we should have emphasized that the main challenge with the supposed ‘peer review’ predictor was its conceptual ambiguity and misalignment. We only realized that the items were deficient during the exploratory analyses, and this was essentially why we discarded it. We will address this further in the response to comment 9 below on the “peer review predictor”.
- Consequently, to better clarify the deviations from the preregistration, we have now extended our description outline the main deviations, emphasizing once more that this is a comprehensive exploratory study. We have included the following overall description in the Methods section, page 10 [manus with track-changes]:
  “The questionnaire is comprehensive and potentially encompasses several studies. While our project is inherently descriptive and exploratory, we initially chose to preregister to reflect our commitment to transparency (https://osf.io/75g3d). However, in hindsight our broad preregistration does not align particularly well with the narrow focus of this exploratory study. Developments and circumstances, therefore, have led to six deviations from what was originally outlined. One, we do not report outcomes according to main fields research. As one of our reviewers pointed out, aggregated analyses are hardly valid if respondents are not exposed to potentially the same set of QRP statements. This issue arises when grouping by main research fields, leading us to remove these results. Two, for clarity, we have reduced the number of QRP categories from twelve to eleven. Three, the scope of this study limits us to addressing only two of the four preregistered outcome variables: self-reported use and perceived prevalence. Four, we discarded the results from the list experiment because the implementation turned out to be deficient in several respects, which we cannot adequately explain. The decision is supported by similar challenges faced in other surveys on integrity [103]. Five, we changed the reported regression model to one not preregistered to ease interpretation, noting that results under the preregistered model are similar. Sixth, we discarded a set of four items from the questionnaire that were originally intended to capture perceptions of peer review and prestigious publication outlets, along with their potential link to QRPs, to explore them as a predictive systemic factor. Unfortunately, these items and their formulations turned out to be problematic for this purpose, resulting in conceptual ambiguity and misalignment with the two other systemic factors included (see survey instrument section below for further elaboration).”

**[6] Medium: Introduction and comparison studies.** The review of the literature is extensive, but the most important part of it, in my opinion, are the several studies that tried to estimate the use and prevalence of QRPs, and to which the authors frequently compare their results. Most readers will not be able to recall the information for all these studies, and the fact that references are numeric instead of in APA format (which would include at least the first author's last name and year of publication), made it very hard for me to keep the comparison studies even vaguely in my head as I read the results. One solution that would substantially improve these comparisons (and the introduction) is to include a table somewhere that summarizes the main studies used in comparisons. The table could include things like authors’ names, the sample size, other relevant sample characteristics, the QRPs that were studied, how the use and/or prevalence was elicited, and even the main results/estimates. If that's too much information to include in a single table, a full table is maybe more appropriate for the supplement or OSF. But even a list of which studies the text is referring to and basic characteristics of their methods would go a long way in helping the reader make sense of the comparisons made in the results and discussion sections.

**Response:**

- We have now included comprehensive supplementary material (S3 Files) that lists the studies we compare our 25 self-reported findings to. We list the reference number used in the manuscript, the full reference, the field surveyed, the sample size(s) if deducible, the number of practices examined, whether the set of practices is broad or narrow (narrow implies the use of similar items to John et al. 2012), and which statements we deem comparable to our items and their estimates.
- Page 23, line 19: We have included the following “and S3 File for a comprehensive summary of surveys we compare our findings to below)”.

**[7] Medium: Preferred research approach**. The authors report that the respondents chose “one preferred research approach among four”—non-empirical, empirical quantitative, empirical quantitative using significance testing, and empirical qualitative—but the survey shows that respondents were funneled into these 4 categories by 3 sequential forced-choice questions: empirical vs. non-empirical approach, quantitative vs. qualitative methods, and a final question, “Are statistical significance tests used in your field of research?”, which is not about whether the researcher themselves employs a quantitative approach using statistical significance tests. “Empirical quantitative” and “empirical quantitative using significance testing” are nested categories and don’t quite reflect the questions respondents were asked, making it more difficult to interpret the findings. I would suggest describing the survey questions in a bit more detail and relabeling the categories accordingly, at least the first time the category is mentioned (e.g., “a researcher that uses primarily empirical, quantitative approaches in a field that uses/doesn’t use significance testing”, which would then not be overlapping categories). Since these categories are, then, not exclusively about the researchers’ methods nor about their fields’ methods, it’s important to describe the distribution of these 4 categories into the researchers’ fields. This will probably be more practical as a figure than a table. (I would suggest that the authors consider making tables 1 and 2 into bar charts as well so the numbers in those tables are more easily comparable.)

- We thank Reviewer 1 for pointing out this inaccuracy in our reporting.
- We have now addressed the issue in more detail and have provided a new figure (Fig 1) that exemplifies the process, as well as outlining which QRP statements are eligible for the four individual approaches (see also response to comment 4 above).

**[8] Medium: Ad hoc predictor scales**. Using ad hoc scales is not in itself a problem, but it would help if the authors presented a little bit more information on these measures in the main manuscript, especially since they make a decision to omit the preregistered peer review measure entirely (but see below). For example, in the Survey Instrument section, when describing the scales, the authors should include a little more explanation of the constructs they are trying to capture (e.g., what does “local research culture” mean?), an example item from each scale, and some information about the reliability and validity of each measure (e.g., alpha or omega coefficients, maybe fit information for a 1-factor CFA model?).

**Response:**

- We have revised the Survey instrument section so that now it contains more thorough motivations for the factors (see also comment 9 below), Survey instrument section, page 17 [manus with track-changes]:
  “These [perceived pressure’ and ‘local research culture’] directly relate to conditions that might compel researchers to compromise integrity. Both ‘perceived pressure’ and ‘local research culture’ have high predictive relevance since external and internal pressures are often cited as drivers of QRPs, making ‘perceived pressure’ likely to be a significant predictor of their use. Conversely, a supportive research culture is logically associated with lower incidences of QRPs, making this factor a plausible protective factor against them.”
- We have downplayed the EFA and provided a thorough explanation for why we eventually discarded the ‘peer review’ factor (see response to comment 9).

**[9] The missing peer review predictor.** Without more information about each measure, it’s hard to know why exactly the authors chose to omit the peer review predictor, but they do mention running an exploratory factor analysis and finding that “only two constructs were valid as scales”. I found this confusing; these are all pretty different constructs, and I’m not sure what putting them all together in an EFA would be able to tell us. I’d much rather get some reliability and fit information for a 1-factor CFA on each ad hoc scale, as mentioned above. If that shows that the peer review items have terrible reliability, is one of the items mainly responsible for the problems? The authors could just drop it, explain what happened, and use the variable as preregistered

**Response:**

- We agree with Reviewer 1 that the arguments for exclusion of the so-called ‘peer review’ predictor from the study are wanting, especially given its initial preregistration.
- The exclusion was primarily due to its conceptual misalignment, realized only after exploratory analyses. Initially, we aimed to examine systemic factors influencing research integrity, including ‘perceived pressure’ and ‘local research culture’. We also thought it useful to include a potential third factor that was supposed to link perceptions of peer review and publication culture to QRP use. However, it became evident that the set of items we formulated and the construct we had envisioned didn’t work for this purpose. The item formulations and the construct turned out to be ill-conceived, generating ambiguity, conceptual misalignment, and problems of interpretation which we unfortunately only realized during our exploratory work. We should therefore not have preregistered this predictor to begin with.
- We recognized that the ‘peer review’ predictor was conceptually misaligned with the two other systemic predictors ‘local research culture’ and ‘perceived pressure’. Unlike the latter two, which directly relate to respondents’ perceptions of their research conditions and how these might influence engagement in QRPs (or serve as justifications for such engagement), the supposed ‘peer review’ predictor primarily captured broader sentiments or instinctive reactions regarding the role of peer review and prestigious outlets in either countering or facilitating QRPs. Thus, it reflects general sentiments on systemic issues within academic publishing rather than specific research conditions influencing QRP engagement.
- The conceptual coherence of the ‘peer review’ predictor is compromised by the mixed nature of its items. These items blend general attitudes towards both the positive and negative impacts of ‘peer review’ and ‘publication in leading outlets’ on research integrity and QRPs, as well as asking respondents directly about their own preference for a QRPs. This introduces risks of social desirability or other forms of response bias, rather than revealing personal inclinations towards QRPs.
- Consequently, including this predictor would lead to diminished conceptual clarity and uncertain predictive relevance, as it tends to capture general sentiments rather than specific conditions directly conducive to QRP engagement. This complexity limits the predictor’s utility in the model, suggesting a relationship with QRPs that is indirect and not as straightforward as with the other two predictors.
- In summary, while the ‘pressure’ and ‘local research culture’ predictors demonstrate direct and conceptually clear connections to self-reported QRP use, the link between the ‘peer review’ factor and QRPs is indirect, complex, and less intuitive, compromising its value in predicting QRP engagement.
- We agree with Reviewer 1 that we should not have put all these items into an EFA to begin with. We should simply have explained that we discarded the ‘peer review’ predictor based on its conceptual ambiguity and misalignment with the other two constructs.
- In order to be transparent, we have now provided the following arguments for why we have discarded the preregistered ‘peer review’ factor in the Survey instrument section, page 17 [manus with track-changes]:
  “However, the 'peer review' factor was eventually discarded. Upon deeper analysis, we realized that the items were not well-suited for the intended purpose. As designed, the 'peer review' factor does not align as directly with the perceived mechanisms influencing QRP engagement as 'perceived pressure' and 'local research culture' do. It presents an indirect and complex relationship with QRPs compared to the direct, clear connections of the other two. These directly relate to conditions that might compel researchers to compromise integrity. Both 'perceived pressure' and 'local research culture' have high predictive relevance since external and internal pressures are often cited as drivers of QRPs, making 'perceived pressure' likely to be a significant predictor of their use. Conversely, a supportive research culture is logically associated with lower incidences of QRPs, making this factor a plausible protective factor against them. While peer review and intense publication competition may indeed compromise integrity, the manner in which the items were formulated—capturing broad sentiments rather than specific proclivities for, or behavioral inclinations towards, QRP engagement—ultimately forfeited the opportunity to accurately reflect that. Thus, including it would compromise the interpretive clarity and predictive relevance of the model.”

**[10] Minor: Were PhD students excluded?** It sounds like having completed a PhD was a prerequisite to participating in this study, which is fine, but it would be good to see some more explanation of why this decision was made, and maybe mention this somewhere in the discussion. There may be relevant differences between researchers who have a PhD and those still in training in terms of QRPs and the other variables measured.

**Response:**

- We have included this explanation in the Survey participants section, page 16-17 line 16-17 [manus with track-changes]:
  “PhD-students were not included as we wanted to ensure that respondents had some” recent publication experiences.”
- First, of all, our approach required that respondents had a record of some recent publications, excluding many PhD-students.
- Secondary, as other analyses had already focused on the difference between students and more senior colleagues, we decided to not pursue this in the current study.

**[11] Minor: Add a bit more guidance on the Bayesian stats.** It would help readers who are not as familiar with Bayesian stats if the manuscript includes a bit more hand-holding when referring to these models. For example, it says “Jeffrey’s prior, (, = 0.5,0.5)” was used, but why was that the case? What does this prior imply or why is it more adequate than other priors? I don’t mean that Bayesian researchers should always include a “primer” with their manuscripts just because the majority of researchers insists on being frequentists, but some extra bit of explanation for the uninformed like me is always greatly appreciated :)

**Response:**

- We have now provided some arguments for choice of priors, both for the analyses of individual QRPs and the models, Analyses subsection, page 20-21 [manus with track-changes]:
- “using weakly informative priors, N(0,1). This prior is balanced. It does dampen unreasonable parameter values but is not so strong as to rule out values that might make sense. Point estimates are reported as medians with 95% quantile posterior intervals (QI). Descriptive model statistics are provided in S17S18-20 19 Tables in S2 file. Finally, we report intervals around self-reported use, prevalence and perceived prevalence estimates (Figs 2-4) using Bayesian highest probability densities (hpd) computed with R binom package (v.1.1-1) using Jeffrey’s prior, Beta(α,β=0.5,0.5). Jeffrey’s prior is a non-informative prior that ensures the posterior distribution is invariant under reparameterization. Additionally, it naturally accommodates the variability inherent in estimating proportions, leading to more robust and reliable posterior estimates, especially in cases with limited or sparse data. As we examine 25 different QRPs we prefer this more objective approach providing conservative estimates ensuring that our results are data-driven.”
- We have also provided an exemplary interpretation of the models and their quantile interval, in the Predictors of prevalence section on page 32 [manus with track-changes]:
  “In Bayesian terms for the non-empirical approach, given the model and the prior, a one z-score increase in ‘perceived pressure’ is associated with an increase in the probability of self-reporting QRP use by 2 percentage points. The 95% quantile interval suggests that, based on our model and prior information, there is a 95% probability that the parameter lies between 0.8 and 3.1 percentage points, with the highest probability density around the median 2 points; and so forth for the interpretation of the other estimates.”

**[12] The way QRP prevalence is calculated was a bit confusing**. I had to go back a few times and I’m not totally sure I understand it now. Maybe an example would help?

**Response:**

- We have now provided an example in the Measure section, page 18-19 [manus with track-changes], where the calculation of prevalence is exemplified:
  “For example, for a certain QRP, 20% of respondents admitted used. Their mean repetition rate is calculated from the non-zero integers on the rating scale (1-6), let’s say the mean is 1.9. We transform 1.9 to a percentage using POMP $\left( \frac{(1.9-0)}{(6-0)} \right)\times100=32.3\%$, and finally we multiply the proportion of non-zero responses (20%) with the mean repetition rate (32.3%) and the self-reported prevalence for the QRP is then 6.5%. “

**[13] Minor: More headings please.** My final point is more of a stylistic suggestion, but I found it very hard to follow the flow of the paper without more subheadings, especially in the results section. Maybe it would help to move some of the information there to the discussion as well, but I just found it hard to follow the results without more headings, especially for a study with so many variables and multiple whole-page figures. I kept losing my place in the manuscript while I looked at the figures, and a few extra subheadings would probably have helped.

**Response:**

- We have rewritten the results section due to the removal of the aggregate results as suggested, as well as the changes to the predictor analyses. We have also included a third level of sub-headings across the whole manuscript which hopefully will make it more accessible.

# Responses to Reviewer 2

We would like to thank Reviewer 1 for the relevant and incisive comments. We have attempted to address them to the best of our ability below.

[1] Minor: I believe that including two or three examples for each of the four main scholarly fields mentioned on lines 14 to 16 of page 11 would help readers to understand better.

**Response:**

- Thanks for this relevant comment. Due to comments from Reviewer 1, all analyses pertaining to main scholarly fields have been removed in this revised manuscript.

[2] Minor: I may be overlooking something, but discussing the generalizability of the study's findings in the discussion or limitations section might be beneficial. In other words, do the findings of this study apply to countries and regions other than Denmark, the UK, the USA, Croatia, and Austria? For instance, the study's participants are primarily researchers from Western countries, but do the authors think similar results would be obtained in Asian countries like Japan? This is merely a personal interest of mine and may be slightly out of the scope of the study, so it is not necessary to address it.

**Response:**

- We thank Reviewer 2 for this relevant comment. We do actually discuss the generalizability in the following sentences in the Implications subsection on page 41 [manus with track-changes]:
  “We therefore argue that these response patterns are very robust, seemingly reproducible despite different settings, and is therefore probably ‘as good as it gets’ from such surveys of QRPs. Note, we do not claim that these are ‘unbiased’ estimates, just reliable response patterns given the instrument.”
