# Supplementary material for: Is something rotten in the state of Denmark? Cross-national evidence for widespread involvement but not systematic use of questionable research practices across all fields of research
Source: PLoS One. 2024 Aug 12;19(8):e0304342. doi: 10.1371/journal.pone.0304342 (PMC11318862; doi:10.1371/journal.pone.0304342)
Supplement: Attachment — Submitted filename: response-to-reviewers.docx [file pone.0304342.s006.docx]

**Response to reviewers**

We want to thank Reviewer 1 and the Editor for further relevant comments. We have attempted to address them to the best of our ability below.

**Editor comment:**

- Please review your reference list to ensure that it is complete and correct. Any changes to the reference list should be mentioned in the rebuttal letter that accompanies your revised manuscript.

**Response**: We have checked the reference list and identified four duplicates (see below), which means that the reference list has been reduced from 113 to 109. This also means that the numbering has changed. We have updated that in the manuscript and in the supplementary files.

| Duplicate references | Old numbers | Revised new number |
| --- | --- | --- |
| Edwards | #29; #30 | #29 |
| Makel | #68; #71 | #68 |
| Bakker | #70; #74 | #69 |
| Chien | #72; #95 | #70 |

**Reviewer 1 comments:**

I have two final suggestions for the discussion section, which I am happy for the authors to incorporate or ignore.

- First comment: First, I still think the citations as numbers makes it a lot harder to understand and remember which study the authors are referring to. The table that was included in the supplement helps with this, but it’s not easily accessible. Instead, I would suggest slightly rephrasing the sentences to include the name of the authors, at least in the discussion if not the whole manuscript. Here’s an example sentence from page 33, lines 10-11, with edits in brackets: “They are also in line with the pooled estimate [obtained by Fanelli] [62], but much higher than the recent estimate [by Xie et al.] [61].”

**Response**: From the results section onwards we indicated the reference compared to in the manner suggested by Reviewer 1.

- Second comment: My second suggestion is to edit the last couple of paragraphs in the paper for a tighter, more effective conclusion — perhaps even adding one last subheading (“Conclusion”) right before the Mazar paragraph? It’s fine as it is and I like the reference to Mazar and colleagues’ claim, it just needs a bit of reworking to be clearer as it’s currently a little difficult to follow.

**Response**: We have rewritten the last paragraphs so that our argument (hopefully) comes through more clearly, and we have also created a ‘Conclusion’ subheading, as suggested.

- **Third comment**: Relatedly, the manuscript has a few typos here and there that the authors might want to address by pasting the final manuscript text into a google doc or some other text-checking tool — it’s certainly nothing that compromises comprehension, though. I only mention this because the PLoS review system says they do not copyedit.

**Response**: We have scrutinized the manuscript for typos and commas and had native English speakers check the grammar. Hopefully, we have identified the most errors and thus made the manuscript more readable.

Again, many thanks for the comments.
